# A late-surviving phytosaur from the northern Atlantic rift reveals climate constraints on Triassic reptile biogeography

**DOI:** 10.1186/s12862-023-02136-8

**Published:** 2023-07-17

**Authors:** Chase Doran Brownstein

**Affiliations:** 1 Department of Ecology and Evolutionary Biology, Yale University, New Haven, CT USA; 2 Stamford Museum and Nature Center, Stamford, CT USA

**Keywords:** Biogeography, Phytosauria, Pangaea, Triassic, Climate change, Phylogenetics

## Abstract

**Background:**

The origins of all major living reptile clades, including the one leading to birds, lie in the Triassic. Following the largest mass extinction in Earth’s history at the end of the Permian, the earliest definite members of the three major living reptile clades, the turtles (Testudines), crocodylians and birds (Archosauria), and lizards, snakes, amphisbaenians, and Tuatara (Lepidosauria) appeared. Recent analyses of the Triassic reptile fossil record suggest that the earliest diversifications in all three of these clades were tightly controlled by abrupt paleoclimate fluctuations and concordant environmental changes. Yet, this has only been preliminarily tested using information from evolutionary trees. Phytosauria consists of superficially crocodylian-like archosaurs that either form the sister to the crown or are the earliest divergence on the crocodylian stem and are present throughout the Triassic, making this clade an excellent test case for examining this biogeographic hypothesis.

**Results:**

Here, I describe a new phytosaur, *Jupijkam paleofluvialis* gen. et sp. nov., from the Late Triassic of Nova Scotia, Canada, which at that time sat in northern Pangaea near the northern terminus of the great central Pangean rift. As one of the northernmost occurrences of Phytosauria, *J. paleofluvialis* provides critical new biogeographic data that enables revised estimations of phytosaur historical biogeography along phylogenies of this clade built under multiple methodologies. Reconstructions of phytosaur historical biogeography based on different phylogenies and biogeographic models suggest that phytosaurs originated in northern Pangaea, spread southward, and then dispersed back northward at least once more during the Late Triassic.

**Conclusions:**

The results presented in this study link phytosaur biogeography to major changes to Triassic global climate and aridity. Together with the earliest dinosaurs and several other reptile lineages, phytosaur diversification and migration appear to have been restricted by the formation and loss of arid belts across the Pangean supercontinent.

**Supplementary Information:**

The online version contains supplementary material available at 10.1186/s12862-023-02136-8.

## Introduction

During the Triassic Period (~ 252–201 million years ago; [35]), reptiles radiated and gave rise to the earliest diversity in the three major living crown clades: turtles, archosaurs, and lepidosaurs (e.g., [8, 10, 42, 46, 52, 68, 69, 82, 83, 92]). Crown reptile faunas that appeared during the Triassic displayed high regional endemism by the end-Triassic mass extinction, despite the persistence of a single supercontinent—Pangaea—to this boundary [14, 27, 48, 70, 80, 90, 93, 94]. Alternative drivers of reptile paleobiogeography have been proposed to explain this seemingly contrasting pair of observations. Most notably, trans-Pangaean dispersals of Triassic reptiles, including dinosaurs and their closest relatives within the Archosauria, may have been tightly regulated by cycles of aridification and intense precipitation (e.g., [4, 20, 21, 25, 81]), which occasionally turned central Pangaea into a superheated desert belt that restricted migration [4, 14, 27, 36].

Phytosauria comprises approximately 35 genera of large, mostly semiaquatic archosauromorphs notable for their longirostrine and often crested skulls (e.g., [12, 15, 16, 22, 23, 41, 47, 85–89]). This clade is almost universally considered to be the earliest divergence in crocodylian-line archosaurs in large-scale phylogenetic analyses of archosauromorphs ([3], 1988; [7, 28, 29, 79, 85]), although at least one analysis has found Phytosauria to be sister to the archosaurian crown [69]. The morphology of phytosaurs (e.g., [12, 39, 78, 85, 89]), the paleoenvironmental settings of many phytosaur specimens [12, 49, 54, 78, 85, 89, 91], and the morphology of tracks suggested to be those of phytosaurs [74] strongly suggest that nearly all phytosaurs were semiaquatic (though see [50]); species in the genus *Mystriosuchus* may have even been seagoing specialists [12, 39, 78].

The fossil record of phytosaurs shows that this clade was distributed across Pangaea (e.g., [85]), and the abundant fossils of different phytosaur species serve as important index fossils for many biochrons in the northern hemisphere [58, 85]. However, this clade was far less common in southern hemisphere, where they are restricted to a handful of occurrences and species [2, 11, 16, 22, 23, 44, 47, 51, 85, 89]. Because phytosaurs appear to have been ecologically tied to watery environments, their biogeography has featured extensively in studies of Pangean reptile dispersal during the dramatic climatic fluctuations that characterized the Triassic [2, 6, 9, 22]. However, the observation that some phytosaurs may have displayed predominately marine [12] or terrestrial [50] ecologies, when considered alongside evidence for the occupation of semiarid paleoenvironments by this clade [2], implies that the dispersal capabilities of this clade may have been less limited than initially supposed.

Incorporating a phylogenetic approach to biogeography allows for a reconstruction of the stepwise manner in which allopatric divergences occurred. Resultantly, phylogenetics provides a powerful tool for interrogating morphological data to test hypotheses about Triassic Pangean biogeography (e.g., [22, 25, 27, 36]). Despite the decades-long interest in phytosaur biogeography and stratigraphy [6, 9, 22, 23, 27, 61, 85, 89], model-based historical biogeographic reconstructions have never been produced for this clade. Further, large geographic gaps remain in the record of phytosaur remains diagnostic to the species level.

Here, I describe a new genus and species of phytosaur from the Late Triassic (Norian-Rhaetian) White Water Member of the Blomidon Formation of Nova Scotia, Canada. *Jupijkam paleofluvialis*, known from a partial skull associated with a single complete osteoderm and numerous bone fragments found in 1974 [90] represents one of the northernmost and geologically youngest occurrences of Phytosauria known to date [2, 22, 85]. Phylogenetic analysis of the new species using multiple morphological matrices and optimality criteria confidently establishes its position outside the clade of latest Triassic phytosaurs that includes robustly-built forms from western North America (*Machaeroprosopus* and kin; e.g., [22, 41, 45, 47, 86–89]). Reconstructions along the tip-dated Bayesian phylogenies generated in this study consistently show that phytosaur dispersal across Pangaea was associated with periods of lower aridity in the center of the continent. In turn, phytosaur phylogeny supports climate change and aridification as major drivers of Triassic vertebrate biogeography across the supercontinent.

## Methods

### Phylogenetic data matrix assembly

The phylogenetic interrelationships of phytosaurs are somewhat controversial. Particularly, different datasets find considerably different relationships for ingroup latest Triassic species [1, 13, 22, 23, 37, 41, 45, 47, 86–89]. In order to properly test the affinities of *Jupijkam paleofluvialis*, I coded this taxon for two recently published matrices. The first, Jones and Butler [45], includes 94 discrete characters coded for 42 ingroup and 1 outgroup operational taxonomic units (OTUs). I modified this matrix so that unnamed OTUs representing particular specimens were removed, as were the two poorly known wildcard species *Phytosaurus doughtyi* and *Paleorhinus parvus.* I removed these species, which could only be coded for a handful of characters, because their incompleteness was likely to negatively impact the accurate resolution of the phylogeny; this is common practice in current phylogenetic analyses of fossil specimens [45, 82, 83]. The removal of these two species and several fragmentary specimen-level OTUs should not affect biogeographic sampling, as all are known from regions extensively represented by the kept sample and indeed might be congeneric with lineages in the kept sample [45]. Next, I added codings for *Jupijkam paleofluvialis* and the Moroccan species *Arganarhinus magnoculus* [26] based on personal observation of the holotypes of both specimens. I also removed *Euparkeria capensis* and instead used *Diandongosuchus fuyuanensis* as the outgroup, as the affinities of the latter species to Phytosauria as the earliest-diverging member of this lineage are now widely accepted (e.g., [29, 31, 89]) and it therefore provides the best outgroup to test the relationships of ingroup phytosaurs (Parasuchidae). *Diandongosuchus fuyuanensis* is known from a complete, articulated skull and skeleton from the Triassic of China and represents the sister to all other phytosaurs as confirmed by multiple analyses (e.g., [29, 31, 89]).

The second dataset I used was that of Datta and Ray [22], which includes 106 discrete characters and 24 OTUs, two of which are unnamed phytosaur species from India. I modified this matrix by removing *Euparkeria capensis* and using *Diandongosuchus fuyuanensis* as the outgroup instead, in addition to adding *Jupijkam paleofluvialis.* Like the Jones and Butler [45] matrix, the Datta and Ray [22] matrix suffers from the disproportional sampling of northern hemisphere phytosaurs, despite the fact that more Indian species are sampled in Datta and Ray [22]. More complete material is needed for phytosaurs from South America (e.g., [51]) and Sub-Saharan Africa (e.g., [2]) to establish species for the phytosaur occurrences in these regions. However, for the time being these matrices represent the most taxon- and character-rich datasets available to reconstruct the relationships and biogeographic history of phytosaurs.

### Parsimony analyses

I conducted parsimony analyses of both datasets in TNT v. 1.5 [34]. In both cases, my methodology was the same. I subjected the matrix to an initial Wagner search over 1000 trees with default settings for ratchet, tree fuse, tree drift, and sectorial search, followed by traditional bisection-reconnection branch swapping over 100,000 trees to explore possible most parsimonious topologies more extensively. The resulting most parsimonious trees were saved and summarized in a strict consensus topology. I calculated and recorded consistency and retention indices and used standard bootstrap values to assess support for particular relationships.

### Bayesian phylogenetic analyses

I conducted Bayesian phylogenetic analyses using the implementation of the Fossilized Birth–Death Model in BEAST 2.6.6 [5, 33] and constructed input files using the BEAUTi terminal. I used the Markov-variable model of character evolution presented in Lewis [55], with characters partitioned by state count. Character partitions are listed in the input files in the Supplementary Data. For the tip dates, I used the age data provided in [45] and several new dates for fossils newly included in both analyses. These include an age for *Jupijkam paleofluvialis* at the Norian-Rhaetian boundary following Gradstein et al. [35] and Sues and Olsen [90], a Carnian-Norian boundary age for the Indian phytosaur species noted in Datta and Ray [22], and a mid-Norian (220.0 Ma) age for *Arganarhinus magnoculus* (see [26, 30]). Finally, an age of 228.5 Ma was used for *Brachysuchus megalodon* from the Dockum Group, which is just older than the boundary of the Carnian and Norian and repressors the midpoint age for Dockum Group phytosaurs [45]. For the clock model, I used a lognormal relaxed clock with a default of 1.0 for the mean and 0.33 for the standard deviation. The diversification rate prior was set to 1 with an allowed range of 0 to infinity, and the origin prior was set to 247.2 million years ago (the base of the Anisian) with bounds of 242.0 (the Anisian-Ladinian boundary, representing the oldest possible age of the latest Ladinian-earliest Carnian phytosaur *Diandongosuchus fuyuanensis*) and 251.9 (the Permian–Triassic boundary) million years ago. *Diandongosuchus fuyuanensis* was set as the outgroup using a monophyletic MRCA prior. I ran two independent runs over 1.0 × 10^8^ generations with 1.0 × 10^7^ pre-burnin, combined the two posterior tree sets in LogCombiner v. 2.6.6 with 10% burnin [5] after checking for convergence of the posteriors using Tracer v. 1.7.1. [76] and summarized the posterior trees in a maximum clade credibility tree with median node heights using TreeAnnotator v. 2.6.4 [5].

### Biogeographic reconstructions

I conducted historical biogeographic reconstructions using the R package BioGeoBears [62] along the maximum clade credibility trees generated using both matrices with geographic distribution data for six regions representing northern (eastern and western North America, Europe, eastern Asia, northern Africa) and southern (India) Pangaea collected from the literature [22, 45, 85]. Three different models, Dispersal-Extinction Cladogenesis (DEC), Dispersal-Vicariance Analysis-like (DIVALIKE), and Bayesian estimation (BAYAREALIKE) were used with and without the j parameter, which allows for jump dispersal between regions. I compared model fit using corrected AIC values, with the lowest value indicating the most favored model, and used chi-squared tests to assess whether model iterations with and without the j parameter differed significantly in support.

## Results

### Systematic palaeontology

Diapsida Osborn [73]

Archosauriformes Gauthier et al. [32]

Archosauria Cope [19]

Phytosauria Jaeger [43]

Parasuchidae Kammerer et al. [47]

*Jupijkam* gen. nov. LSID: urn:lsid:zoobank.org:act:644BCFB8-7EE3-42E7-8F38-C8DAAE82D3C5

### Etymology

After the horned serpent (also Jipijka’m, Chepechcalm, Tcipitckaam; see http://www.native-languages.org/jipijkam.htm) of Mi’kmaq tradition; the Mi’kmaq are the First Nations people from Nova Scotia and adjacent areas of the Northern Woodlands.

### Diagnosis

Same as for the type and only known species.

*paleofluvialis* sp. nov. LSID: urn:lsid:zoobank.org:act:E1DC8A3B-DAD6-45F3-8E27-E2BA6E618CC3.

### Etymology

From the Latin paleo (ancient) + fluvialis (from/of rivers, fluvial), in reference to the likely habitat and paleoenvironmental context of this species.

### Material

**Fig. 1:**
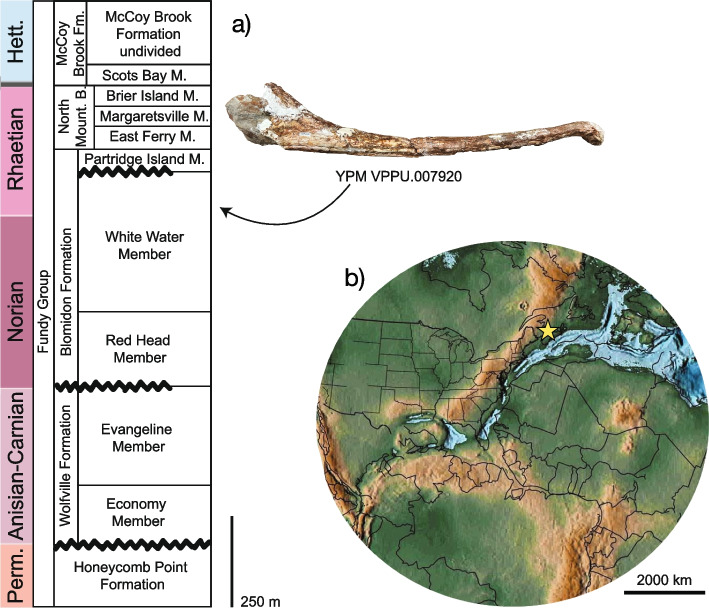
Locality and horizon of the new phytosaur. Stratigraphic column (**a**) of Triassic units in the Bay of Fundy region of Nova Scotia, showing the horizon from which the holotype of *Jupijkam paleofluvialis* was recovered, after Sues and Olsen [90]. Location (**b**) of the site of discovery of the holotype placed on a global paleogeographic reconstruction by C. Scotese in GPlates

**Fig. 2:**
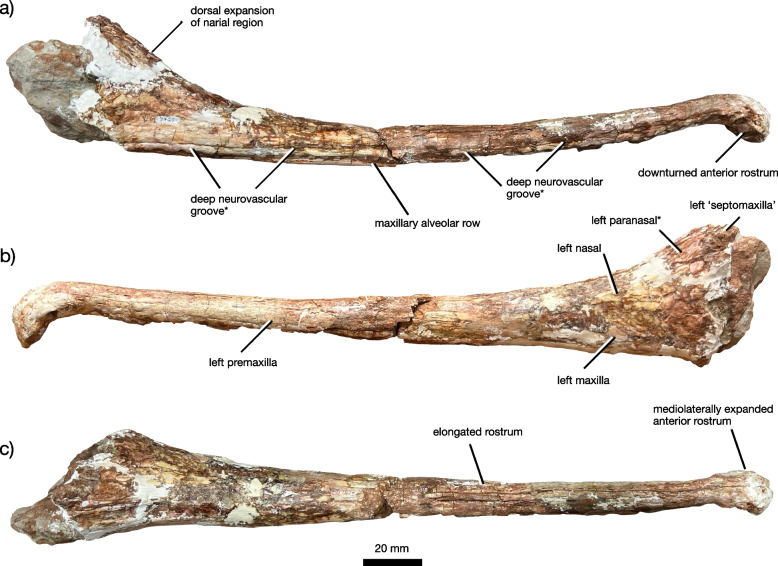
The partial skull of *Jupijkam paleofluvialis* gen. et sp. nov. Preorbital skull YPM VPPU 7920 in (**a**) right lateral, (**b**) left lateral, and (**c**) dorsal views

**Fig. 3:**
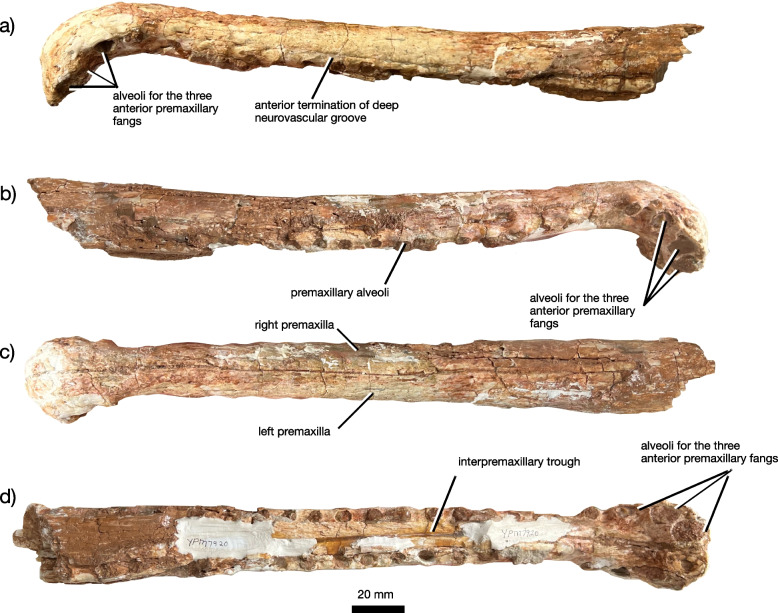
The anterior rostrum of *Jupijkam paleofluvialis* gen. et sp. nov. Anterior rostrum portion of YPM VPPU 7920 in (**a**) left lateral, (**b**) right lateral, (**c**) dorsal, and (**d**) ventral views

**Fig. 4:**
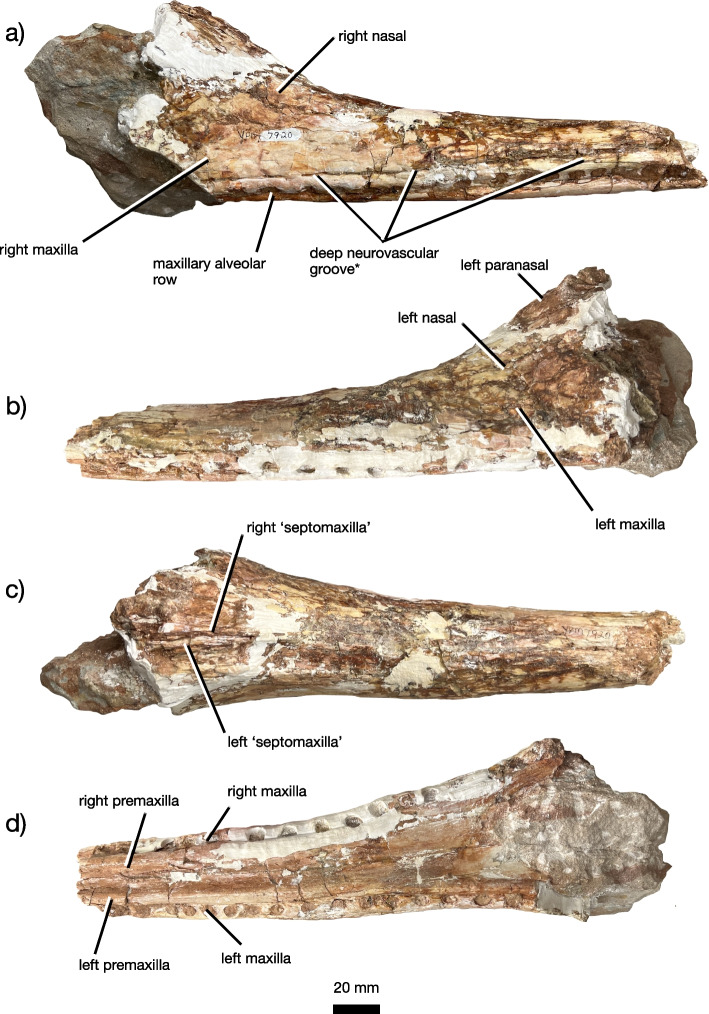
The posterior rostrum of *Jupijkam paleofluvialis* gen. et sp. nov. Posterior rostrum portion of YPM VPPU 7920 in (**a**) left lateral, (**b**) right lateral, (**c**) dorsal, and (**d**) ventral views

**Fig. 5:**
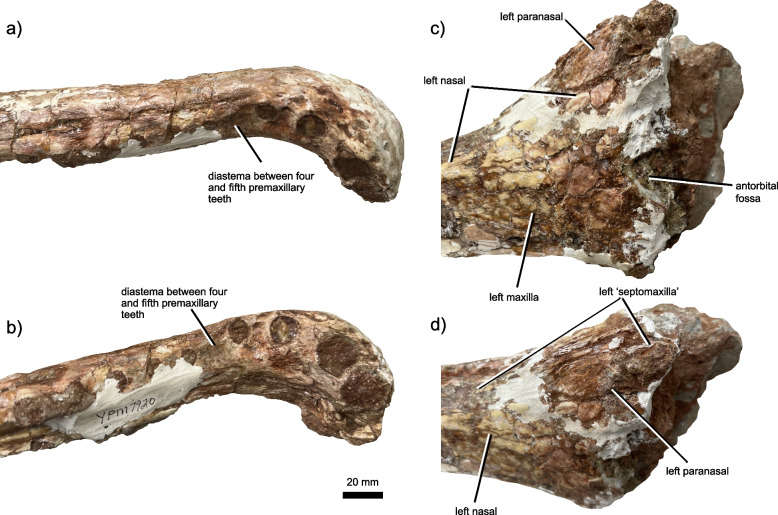
Additional cranial anatomy of *Jupijkam paleofluvialis* gen. et sp. nov. Anterior end of rostrum of YPM VPPU 7920 in (**a**) right lateral and (**b**) right lateral-ventral oblique views. Prenarial region of YPM VPPU 7920 in (**c**) left lateral and (**d**) left lateral-dorsal oblique views

**Fig. 6:**
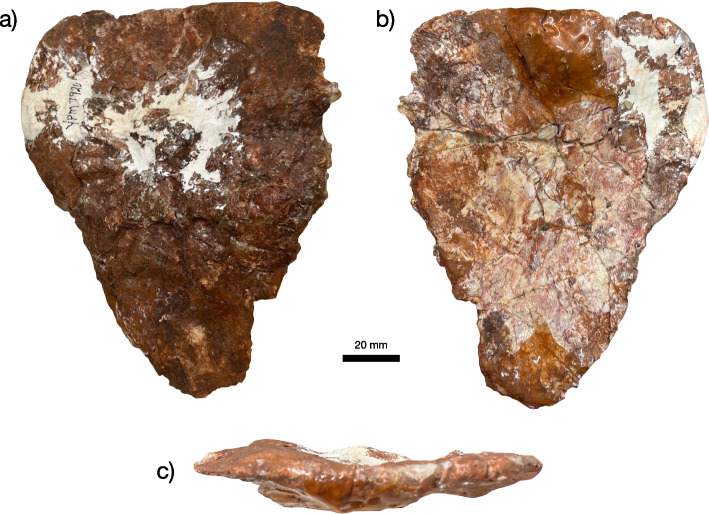
Osteoderm of *Jupijkam paleofluvialis* gen. et sp. nov. Osteoderm of YPM VPPU 7920 in (**a**) dorsal, (**b**) ventral, and (**c**) lateral views

**Fig. 7:**
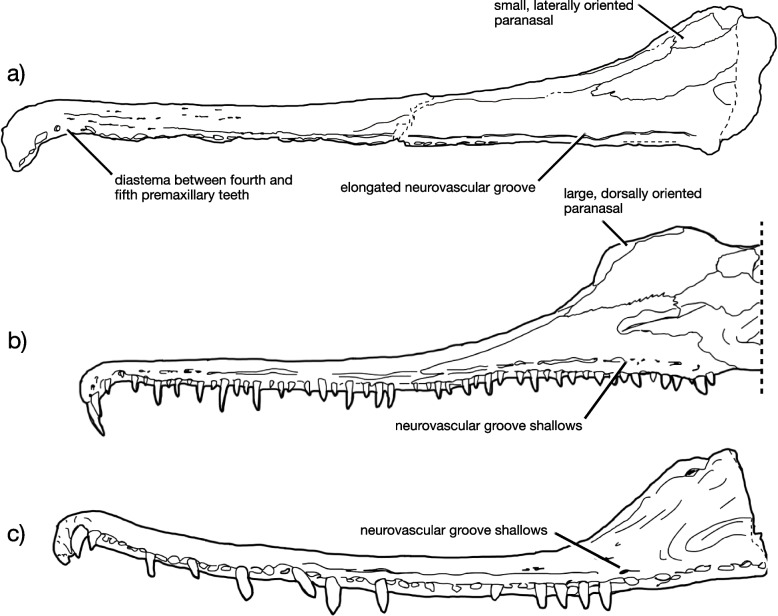
Comparative anatomy of *Jupijkam paleofluvialis* gen. et sp. nov. Line drawings of (**a**) the holotype skull of *Jupijkam paleofluvialis* YPM VPPU 7920, (**b**) the skull of *Rutiodon carolinensis* USNM VP5373 after Colbert [17], and (**c**) the holotype skull of *Machaeroprosopus lottorum* TTU-P10076 after Hungerbühler et al. [41], all in left lateral view. The groove in (**a**) has been reconstructed based on the right side

Princeton University Vertebrate Paleontology Collections acquired by the Yale Peabody Museum (YPM VPPU) 7920 (Figs. 1, 2, 3, 4, 5, 6 and 7), a nearly complete antorbital skull, a closely associated osteoderm, and numerous bone fragments recovered from the Bay of Fundy, Nova Scotia, Canada in 1974 by A. Heimlich, B. Salvia, and P. E. Olsen. The specimen has been stabilized using white plaster that clearly differs in color from the bone (Fig. 2), but images of the specimen in the field [90] show that the shape is not changed by these stabilization measures (~ 90% is bone). Care was taken during examination to disregard artifacts of stabilization.

### Locality and horizon

The holotype specimen of *Jupijkam paleofluvialis* was recovered from the White Water Member of the Blomidon Formation (Fig. 1a) in Series III of the Bay of Fundy Triassic strata as defined by Sues and Olsen [90]. The White Water Member overlies the coarse-grained Red Head Member and underlies the Partridge Island Member, which is the lowest portion of Series IV [90]. YPM VPPU 7920 is the only substantial reptile body fossil recovered to date from the White Water Member, which consists of rhythmic sediments called sand-bank cycles [84] that are much more conducive to the preservation of ichnofossils [90]. Together with phytosaur remains from the Fleming Fjord Group of Greenland [57], YPM VPPU 7920 is the northernmost occurrence of a phytosaur in the Americas and adjacent islands; it is also the only example of this clade from Canada [90]. In the Norian-Rhaetian, the locality of origin for YPM VPPU 7920 sat towards the northern terminus of the great central Pangean rift system (Fig. 1b).

### Diagnosis

Large-bodied, extremely longirostrine parasuchid phytosaur with the following autapomorphies: primary neurovascular foramina row on skull is deeply invaginated to form an anteroposteriorly-running groove along the lateral surface of the rostrum that terminates posterior of the anterior corner of the antorbital fenestra; large diastema between the four anterior premaxillary fangs and the rest of the tooth row; no ridges or other extensive ornamentation on rostrum besides occasional foramina; paranasal faces laterally; nasal dorsoventrally uninflated.

### Description

#### General skull morphology

Only the rostrum and the raised preorbital region of the skull are included in the holotype (Figs. 2, 3, 4, 5 and 6). The rostrum is highly elongated, comparing favorably to forms such as *Rutiodon carolinensis* [17, 18, 24], *Machaeroprosopus lottorum* [41], and *Mystriosuchus* spp. (e.g., [12, 39]), *Angistorhinus grandis* [67], and *Parasuchus* spp. [16, 47], but differing considerably from most robustly-skulled *Machaeroprosopus* (see [45, 85]), *Colossosuchus techniensis* [22], and *Pravusuchus hortus* [86]. The nasal region is distinctly raised relative to the rest of the skull. The antorbital fenestra anterior border sits anterior to the anterior border of the external nares and is recessed in a small antorbital fossa. Measurements of the skull are included in Table 1.

**Table 1:** Measurements of *Jupijkam paleofluvialis* holotype YPM VPPU.007920

Element	Measurements (mm)
Skull anterior portion length	370
Skull anterior portion width	44 (anterior tip), 25 (diastema), 38 (midpoint)
Skull posterior portion length	340
Skull posterior portion width	42 (midpoint), 92 (posterior end)
Skull posterior portion depth	120
Scute	130 (maximum length), 99 (maximum width)

#### Premaxilla

The paired premaxillae (Figs. 2, 3, 4 and 5) are elongated, tubular elements that together form most of the rostrum and antorbital skull. The anterior rosette composes the expanded anteriormost portion of the premaxillae. Each premaxilla houses four alveoli along either portion of the anterior rosette, which is moderately downturned in comparison to *Colossosuchus techniensis* and some species of *Machaeroprosopus* but similar to most other phytosaurs [22]. As in other parasuchid phytosaurs, the four anteriormost alveoli (Fig. 3) show great size differences such that the first three are greatly enlarged relative to the fourth [22, 38]. The first two of these teeth are placed along the anterior margin of the rostrum, and together with the third and fourth teeth are separated from the rest of the premaxillary row by a diastema, which also causes the anterior rosette to appear pinched off from the rest of the premaxillae in dorsal and ventral views (Figs. 3 and 5a-b). Posteriorly, the premaxillae are tubular and do not bear a premaxillary crest. Ventrally, the premaxillae each bear 19 premaxillary teeth posterior to the anterior rosette, all of which are housed in elliptical alveoli that are widely spaced relative to those in most longirostrine phytosaurs, including *Rutiodon carolinensis* [17, 18, 24], *Machaeroprosopus lottorum* [41], *M. pristinus* (e.g., [85]), and *Angistorhinus grandis* [67]. The alveolar ridges, which medially border the alveoli, are ventrally rounded and form the external borders of the long and slender interpremaxillary fossa. Laterally, each premaxilla bears a deeply invaginated groove approximately 5 mm above the alveolar margin that represents the modified primary neurovascular foramina row. In *Machaeroprosopus lottorum* [41] and *M. pristinus* (e.g., [85]), the neurovascular foramina row is also partially developed into a groove, but it is not nearly as deeply invaginated as in *Jupijkam paleofluvialis* nor does it run continuously along both the entire surface of the premaxilla and maxilla to terminate behind the level of the anterior border of the antorbital fenestra (Fig. 4). Posteriorly, the premaxillae articulate with the maxillae and nasals along a gently anteroventrally-sloping suture line. These bones are tightly articulated such that the suture is poorly visible. Due to damage to the specimen, the shape of the articulations between the premaxillae, ‘septomaxillae,’ and paranasals are somewhat unclear. Each premaxilla bore a thin dorsal process in this region that would have articulated with the paranasal laterally and ‘septomaxilla’ posteriorly (Figs. 3 and 5c-d).

#### Maxilla

The maxilla is a triradiate bone that composes approximately half of the lateral posterior section of the preorbital skull along with the nasal (Figs. 2, 4 and 5). Together with the premaxilla and nasal, the maxilla forms an interdigitated suture line that gradually trends anteroventrally along the lateral margin of the preorbital skull. The dorsal margin of the maxilla weakly bulges in the region where the premaxilla, maxilla, and nasal all articulate. Unlike species of *Machaeroprosopus* except for *M. lottorum* [41], the nasal-maxilla suture is relatively straight. This suture is extremely tight and partially ablated. The maxilla of *Jupijkam paleofluvialis* also lacks the distinctive maxillary crests present in *Colossosuchus techniensis* [22]. Posteriorly, the maxilla forms a large portion of the border of the antorbital fenestra. The angle of incline of the maxilla ascending process is comparable to those of *Rutiodon carolinensis*, *Parasuchus hislopi*, and *Wannia scurriensis* among longirostrine phytosaurs, but differs from the much more strongly inclined condition in *Angistorhinus grandis* and *Machaeroprosopus lottorum* [22, 23, 41]. The maxilla bone surface anterior and ventral to the antorbital fenestra is recessed to form an antorbital fossa. Ventrally, the maxilla and premaxilla articulate posteromedial and anterior to the first maxillary alveolus. The maxillary alveolar ridges are not prominent. Posteriorly, the ventral surfaces of the maxilla expand medially. However, the precise articulations of the premaxillae, maxillae, and palatal bones surrounding the choana are unclear.

#### ‘Septomaxilla’

The ‘septomaxillae’ (see [86] for a discussion of the issue of homology surrounding the ‘septomaxillae’ in phytosaurs) are paired elements that form a portion of the dorsal medial region of the posterior prenasal skull and the majority of the internarial septum (Figs. 4 and 5). Anterior to the naris, the ‘septomaxillae’ each bear an elongated premaxillary process that extend approximately the same distance anteriorly as the nasals. The premaxillary process of each ‘septomaxilla’ is straight, rather than laterally concave as in longirostrine *Machaeroprosopus* [41]. Unlike many phytosaurs (e.g., [22, 41, 85, 86]) with the notable exceptions of *Mystriosuchus* spp. (e.g., [12]), *Rutiodon carolinensis* [17, 18, 24], and *Volcanosuchus statisticae* [23], the ‘septomaxillae’ are not extensively ornamented with asymmetrical grooves and ridges. The ‘septomaxillae’ are not strongly arched dorsally as in *Pravusuchus hortus* [86] and *Angistorhinus grandis* [67]. The ‘septomaxillae’ each widen mediolaterally towards the posterior end of the preserved portion of the skull. These bones articulate with the paranasals along the lateral margins of their preserved posterior halves.

#### Paranasal

A thick, subovoid bone placed along the anterolateral margin of the left external naris (Figs. 2, 4 and 5) compares favorably with the paranasal of Hungerbühler et al. [41]. The anatomy of the anterior margin of the naris and the homology of the ossifications of this region with the bones of the skull in other archosauromorphs is somewhat uclear (e.g., [39, 41, 86]). However, a similarly thick, elliptical ossification at the anteriormost point of the naris is not present in any species of *Parasuchus* (see [47]), *Mystriosuchus* [12, 41], *Paleorhinus* [13], *Ebrachosuchus neukami* [13], *Colossosuchus techniensis* [22], and *Volcanosuchus statisticae* [23]. Hungerbühler et al. [41] hypothesized that the paranasal bone may be widespread in western North American phytosaurs placed in the genus *Machaeroprosopus* (i.e., see [40]. Additionally, the domed lateral portion of the ‘septomaxilla’ in *Pravusuchus hortus* (see [86]) bears resemblance to this ossification. Compared to *Machaeroprosopus lottorum*, the paranasal is placed more laterally on the skull in *Jupijkam paleofluvialis* (Fig. 5).

#### Nasal

The nasals, together with the maxillae, form much of the posterior prenarial and the anterolateral narial region of the skull. The nasal forms a tight, interdigitating suture with the maxilla and anteriorly thins to an apex approximately one third of the length to the anterior terminus of the maxilla (Figs. 2, 4, 5). Dorsally, the nasal abuts the paranasal, the anterior process of the ‘septomaxilla', and presumably the section of the ‘septomaxilla’ posterior to the posterior end of the paranasal. Because only the antorbital portion of the skull of *Jupijkam paleofluvialis* is preserved, the anatomy of the posterior portion of the nasal is unknown.

#### Osteoderms

A single complete osteoderm was preserved closely associated with the holotype skull of *Jupijkam paleofluvialis* (Fig. 6). This osteoderm is large, polygonal, has a poorly developed median boss that is placed centrally, and preserves extensive ornamentation that includes numerous ridges and pits. Together, these features suggest the osteoderm is from a dorsal paramedian row [16, 22].

### Phylogenetic analyses

#### Parsimony analysis of matrix 1

**Fig. 8:**
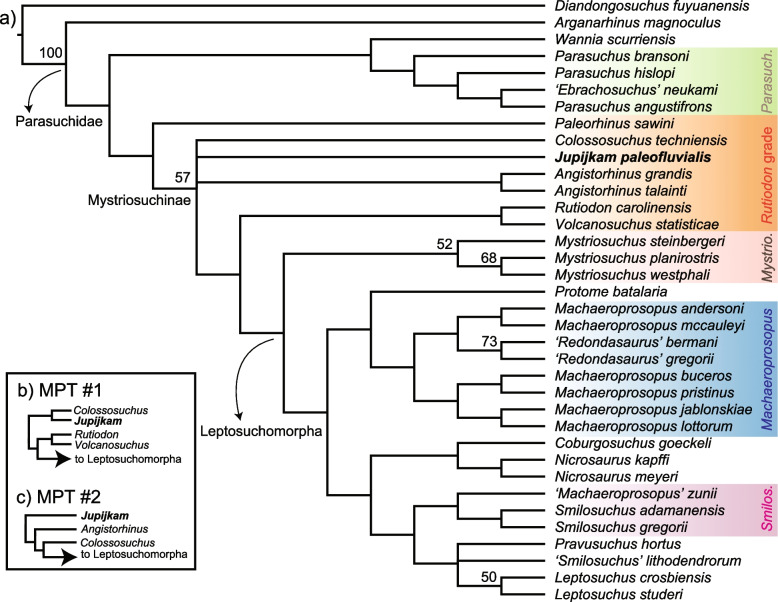
Phylogenetic position of *Jupijkam paleofluvialis* gen. et sp. nov. Strict consensus topology (**a**) and portions of the two most parsimonious trees recovered (**b**-**c**) from the parsimony analysis of the modified dataset of [45]. Numbers at nodes are standard bootstrap supports

The analysis of the modified matrix of [45] (2018) using TNT v. 1.5 [34] produced a strict consensus (Fig. 8a) of 2 most parsimonious trees (Fig. 8b-c), each of length 315 (consistency index = 0.403, retention index = 0.654) that found *Jupijkam paleofluvialis* to be a parasuchid phytosaur of unclear affinities to the major Late Triassic clades in Mystriosuchinae sensu Datta et al. [23]. In the strict consensus tree, *Jupijkam paleofluvialis* forms a polytomy with the Indian species *Colossosuchus techniensis*, the clade consisting of both species of *Angistorhinus*, the clade consisting of *Rutiodon carolinesis* and *Volcanosuchus techniensis*, and the clade formed by phytosaurs in the genera*, Coburgosuchus*, *Leptosuchus*, *Machaeroprosopus, Mystriosuchus*, *Nicrosaurus*, *Pravusuchus*, *Protome*, ‘*Redondasaurus*,’ *Smilosuchus*, and *Leptosuchus*. This position for *Jupijkam paleofluvialis* is supported by the following apomorphies: narial crest rises relatively abruptly (7:1), posterior rim of nares behind anterior rim of antorbital fenestra (14:2), subtriangular antorbital fenestra (based on the preserved portion and comparisons with other phytosaur skulls; 81:2). A complete list of apomorphies of nodes resolved in this analysis is included in the Supplementary Information. The relationships of phytosaurs are weakly supported in this tree. However, the phylogeny recovered in this study generally agrees with that in [45]; *Ebrachosuchus neukami*, *Parasuchus* spp., *Wannia scurriensis*, and ‘*Paleorhinus*’ *sawini* are found to be non-mystriosuchine parasuchids, and *Coburgosuchus goeckeli*, *Leptosuchus studeri*, *Machaeroprosopus* spp.*, Mystriosuchus* spp., *Nicrosaurus* spp., ‘*Redondasaurus*’ spp., and *Smilosuchus* spp. form an ingroup of mostly large-bodied, Late Triassic species. As found in Datta et al. [23], *Volcanosuchus statisticae* and *Rutiodon carolinensis* form a weakly supported clade. The strict consensus topology supports the monophyly of *Angistorhinus*, *Mystriosuchus*, and *Nicrosaurus*, a result also obtained by [45]. The strict consensus topology and the two most parsimonious trees consistently place ‘*Redondasaurus*” deep within *Machaeroprosopus* species, supporting the synonymy of the former genus with the latter (e.g., [41]). *Arganarhinus magnoculus*, newly added to this matrix based on personal observation of the only known skull, is found to be an early-diverging parasuchid sister to all other members of this clade (Fig. 8). However, this placement may be affected by the immature ontogenetic status of this specimen (Fara and [38]).

#### Parsimony analysis of matrix 2

**Fig. 9:**
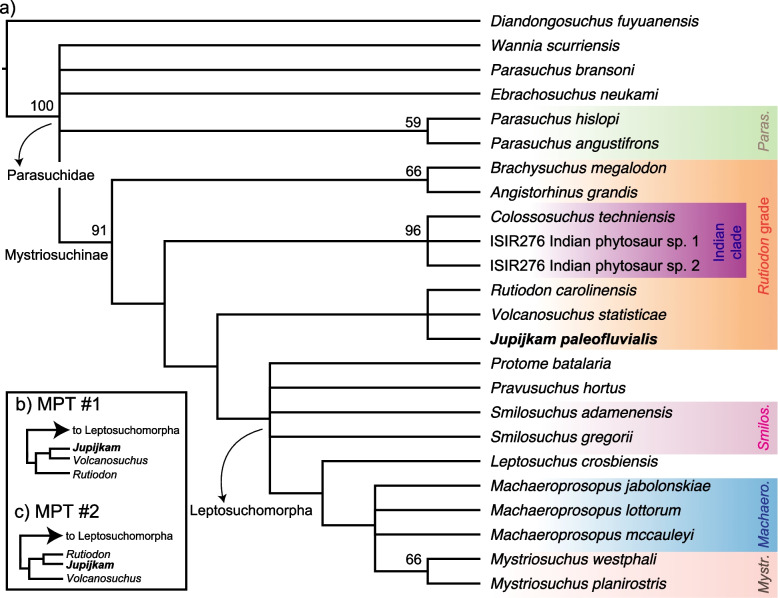
Phylogenetic position of *Jupijkam paleofluvialis* gen. et sp. nov. continued. Strict consensus topology (**a**) and portions of two most parsimonious trees recovered (**b**-**c**) from the parsimony analysis of the modified dataset of Datta and Ray [22]. Numbers at nodes are standard bootstrap supports

The analysis of the second matrix [22] recovered 27 most parsimonious trees of length 349, consistency index of 0.444, and retention index of 0.576. *Jupijkam paleofluvialis* was again found to be a ‘*Rutiodon*’-grade phytosaur (Fig. 9a-c) in a clade with *Rutiodon carolinensis* and *Volcanosuchus techniensis*, which is placed sister to ‘leptosuchomorph’ phytosaurs (see [85, 86]). The position of *Jupijkam paleofluvialis* in the clade containing all phytosaurs more closely related to *Rutiodon* and *Mystriosuchus* than to *Colossosuchus* and the Indian phytosaur radiation described by Datta and Ray [22] is supported by a single character: paramedian osteoderms are roughly circular with a centrally placed boss (103:2). The single complete paramedian osteoderm of *Jupijkam paleofluvialis* possesses a centrally placed boss and the main body is roughly circular. However, this character was only coded as ‘2’ for the two species of *Smilosuchus* included in the original dataset, and so it likely will need to be revised. The position of *Jupijkam paleofluvialis* in a clade with *Rutiodon carolinensis* and *Volcanosuchus techniensis* is supported by two characters: internarial septum composed of more 50% by the ‘septomaxilla’ (9:3) and antorbital fossa present along the anterior or posterior margin of the antorbital fenestra (16:1). The first character is coded in *Jupijkam paleofluvialis* based on the posterior extension of the ‘septomaxillae’ relative to the paranasal and the anterior section of the left naris that is preserved. The second character is coded in *Jupijkam paleofluvialis* based on the presence of the antorbital fossa at the anterior corner of the maxilla, which rapidly reduces in dorsoventral depth posteriorly (Fig. 5c, d). This clade is, however, weakly supported. Indeed, the only ingroup clades that receive moderate to high bootstrap support are (1) Mystriosuchinae, (2) the clade consisting of *Brachysuchus megalodon* and *Angistorhinus grandis*, (3) the Indian phytosaur clade reported by Datta and Ray [22], and (4) *Mystriosuchus* spp.

#### Bayesian analysis of matrix 1

**Fig. 10:**
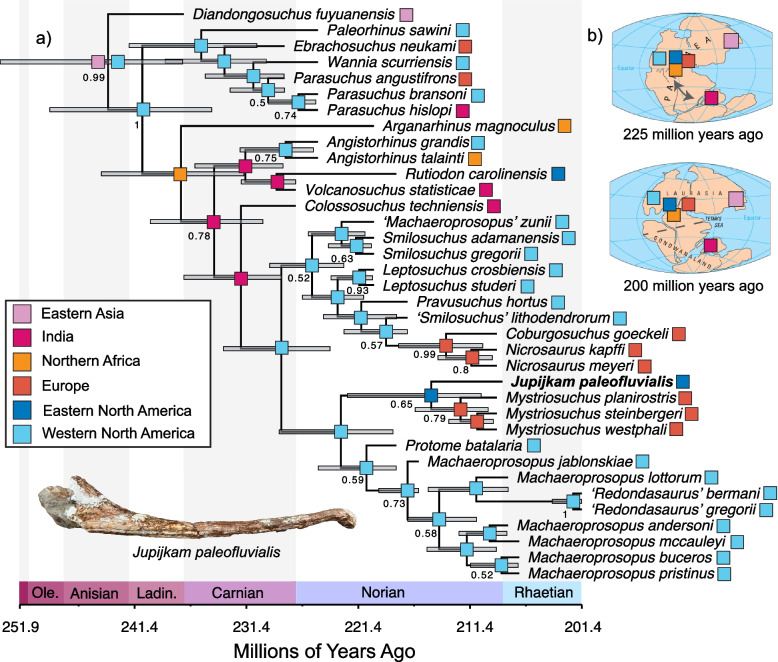
Bayesian tip-dated phylogeny of phytosaurs and biogeographic reconstruction. Maximum clade credibility tree (**a**) resulting from the tip-dated Bayesian analysis of the modified dataset of [45] (2018). Bars at nodes indicate 95% highest posterior density (HPD) intervals for node divergence times, numbers at nodes are posterior support values, and colored boxes represent the ancestral areas estimated using BioGeoBears in R. The panel in (**b**) shows the position of localities sampled in the tree on a simplified map of Pangaea by the United States Geological Survey

Bayesian tip-dating analysis in BEAST 2.6.6 of the first dataset resolved *Jupijkam paleofluvialis* as the sister taxon to *Mystriosuchus* spp., contrasting with the parsimony analyses of both datasets. This position is relatively weakly supported by posterior values, as is most of the topology (Fig. 10). However, as in the parsimony analysis, *Protome batalaria* is resolved as the sister taxon to *Machaeroprosopus* (including ‘*Redondasaurus*’), *Nicrosaurus* spp. is resolved as the sister to *Coburgosuchus goeckeli*, ‘*Machaeroprosopus*’ *zunii* is placed in a clade with *Smilosuchus* spp., *Rutiodon carolinensis* and *Volcanosuchus techniensis* are resolved as sister species, *Angistorhinus* is found to be monophyletic, and *Parasuchus* spp., *Ebrachosuchus neukami*, ‘*Paleorhinus*’ *sawini*, *Wannia scurriensis*, and *Arganarhinus magnoculus* are found to be early-diverging parasuchid phytosaurs. In contrast to the strict consensus tree (Fig. 8), ‘*Paleorhinus*’ *sawini* is found to be the sister taxon to the clade containing *Parasuchus* spp., *Ebrachosuchus neukami*, and *Wannia scurriensis*, *Arganarhinus magnoculus* is found to be the sister species to Mystriosuchinae, *Angistorhinus* spp. is resolved as the sister taxon to the clade containing *Rutiodon carolinensis* and *Volcanosuchus techniensis*, and *Leptosuchus* spp., *Pravusuchus hortus*, and *Smilosuchus* spp. form a grade leading to *Nicrosaurus* and *Coburgosuchus*. The split at the base of Parasuchidae is estimated to occur 240.72 Ma (95% HPD: 234.51, 248.99 Ma), and the split at the base of Mystriosuchinae is estimated to occur 234.29 Ma (95% HPD: 229.92, 239.99 Ma). The clade containing *Jupijkam paleofluvialis* and *Mystriosuchus* spp. diverges from *Protome batalaria* and *Machaeroprosopus* spp. 222.95 Ma (95% HPD: 218.3, 228.46 Ma), and *Jupijkam paleofluvialis* diverges from *Mystriosuchus* 214.87 Ma (95% HPD: 210.41, 222.36 Ma). Divergence times of biogeographic significance include the divergence of the Indian species *Volcanosuchus techniensis* from the eastern North American *Rutiodon carolinensis* 228.71 Ma (95% HPD: 227, 231.86 Ma) and the divergence of *Colossosuchus techniensis* (and presumably the Indian radiation recognized by [22]) from leptosuchomorph phytosaurs 231.89 Ma (95% HPD: 228.24, 237.09 Ma).

#### Bayesian analysis of matrix 2

**Fig. 11:**
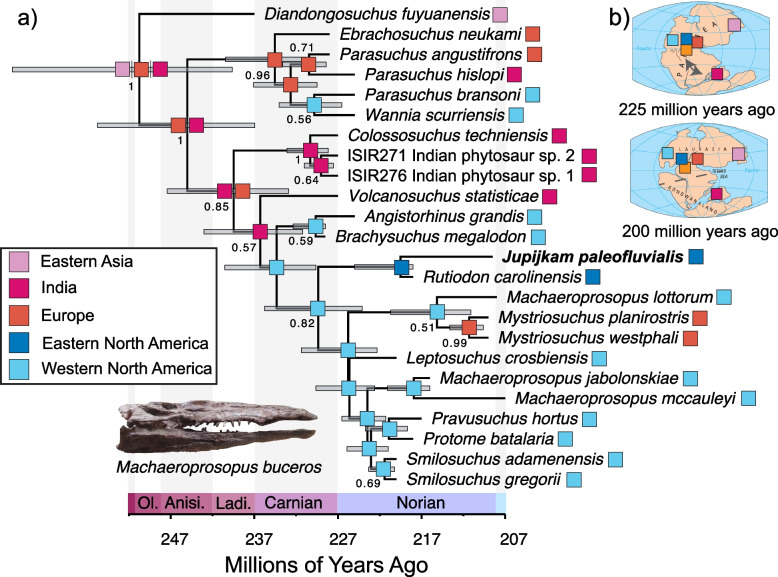
Bayesian tip-dated phylogeny of phytosaurs and biogeographic reconstruction continued. Maximum clade credibility tree (**a**) resulting from the tip-dated Bayesian analysis of the modified dataset of Datta and Ray [22]. Bars at nodes indicate 95% highest posterior density (HPD) intervals for node divergence times, numbers at nodes are posterior support values, and colored boxes represent the ancestral areas estimated using BioGeoBears in R. The panel in (**b**) shows the position of localities sampled in the tree on a simplified map of Pangaea by the United States Geological Survey

The Bayesian tip-dating analysis of the second matrix (modified [22]) recovered a topology that was similar to the strict consensus tree (Fig. 11). *Jupijkam paleofluvialis* is placed as the sister species to *Rutiodon carolinensis* with weak posterior support; this clade is found to be the sister to the clade containing *Mystriosuchus* spp., *Machaeroprosopus* spp., *Leptosuchus crosbyensis*, *Pravusuchus hortus, Protome batalaria,* and *Smilosuchus* spp. Oddly, *Machaeroprosopus lottorum* is found to be the sister species to *Mystriosuchus* spp., a poorly supported result that may be driven by the favored grouping of species of similar ages under the FBD model, rather than strong character support. Indeed, the only strongly supported nodes on this tree are the node subtending the two species of *Mystriosuchus*, the node subtending the Indian phytosaur clade reported by Datta and Ray [22], the early-diverging parasuchid clade containing *Ebrachosuchus neukami*, *Wannia scurriensis*, and *Parasuchus* spp., the clade containing the parasuchid clade exclusive of *Parasuchus* spp., and the clade containing parasuchids exclusive of *Colossosuchus techniensis*. *Jupijkam paleofluvialis* and *Rutiodon carolinensis* are estimated to diverge 219.52 Ma (95% HPD: 218, 225.03 Ma).

### Biogeographic reconstructions

#### Reconstruction along tip-dated tree using matrix 1

Comparison of model fit using BioGeoBears [62] on the Bayesian tip-dated phylogeny produced from analysis of the modified [45] (2018) dataset favored the DIVALIKE model (Table 2), although preference for DIVALIKE over DIVALIKE + j was statistically insignificant. Given that phytosaurs were large, aquatic to partially terrestrial reptiles, I regard jump dispersal as relatively unlikely and show the results of the DIVALIKE construction in Fig. 10a; note that reconstructions of phytosaur historical biogeography under all models implemented were similar.

**Table 2:** Biogeographic model comparisons for the analysis of Matrix 1^a^

Element	Corrected AIC value
DEC	122.8
DEC + j	96.03
DIVALIKE	94.74*
DIVALIKE + j	94.75*
BAYAREALIKE	100.6**
BAYAREALIKE + j	100.6**

^*^P >  > 0.05; no significant difference between -/ + j

^**^ *P* >  > 0.05; no significant difference between -/ + j

^a^Based on Jones and Butler [45]

The DIVALIKE reconstruction favored a northern hemisphere origin (Fig. 10b) for Phytosauria and Parasuchidae, followed by dispersal to the southern hemisphere at the base of phytosaurs exclusive of *Parasuchus* and subsequent return to the northern hemisphere during the Carnian to earliest Norian by three clades, *Angistorhinus* spp., *Rutiodon carolinensis*, and leptosuchomorph mystriosuchines, during the Carnian-Norian. Two European radiations appear during the late Norian: *Mystriosuchus* spp. and *Nicrosaurus* spp. + *Coburgosuchus goeckeli.* Both of these radiations appear from North American clades, implying broad dispersal capability across northern Pangea in the Late Triassic.

#### Reconstruction along tip-dated tree using matrix 2

The DIVALIKE model iterations with and without the jump dispersal parameter j were equally favored (Table 3) in comparisons of model fit for historical biogeographic reconstructions along the tip-dated Bayesian phylogeny from analysis of the modified matrix of Datta and Ray [22]. The DIVALIKE reconstruction, shown in Fig. 11a-b, generally produces less well-resolved ancestral area reconstructions than the historical biogeographic reconstruction under the same model projected on the tip-dated tree made using the modified Jones and Butler [45] dataset. However, several similarities persist, including the restriction of all trans-hemispheric dispersal-vicariance events before the Norian.

**Table 3:** Biogeographic model comparisons for the analysis of Matrix 2^a^

Element	Corrected AIC value
DEC	65.22
DEC + j	55.68
DIVALIKE	54.04*
DIVALIKE + j	54.04*
BAYAREALIKE	60.06**
BAYAREALIKE + j	69.38**

^*^P >  > 0.05; no significant difference between -/ + j

^**^ *P* >  > 0.05; no significant difference between -/ + j

^a^Based on Datta and Ray [22]

## Discussion

### Phytosaur diversity in the latest triassic

The holotype specimen of *Jupijkam paleofluvialis* was collected nearly half a century ago. Formal description of the partial skull and osteoderm of this species allows for a revised understanding of the phylogenetic and morphological diversity of Late Triassic phytosaurs. The geological setting of *Jupijkam paleofluvialis* makes it one of the geologically youngest and northernmost occurrences of the clade Phytosauria ([90]; also see [57]). The minimum age of *Jupijkam paleofluvialis* is the Norian-Rhaetian boundary, meaning that this species is substantially younger than the other eastern North American phytosaur known from diagnostic material: *Rutiodon carolinensis* [17, 90] and indeed most phytosaur diversity (Figs. 10 and 11; e.g., [22, 45, 85]). In the Norian-Rhaetian, phytosaur diversity included the robustly-built *Nicrosaurus* spp. and *Coburgosuchus goeckeli* in Europe and *Machaeroprosopus* spp. (including ‘*Redondasaurus*’) in western North America. *Mystriosuchus* spp. [12, 57] from Europe and Greenland exclusively includes longirostrine species, and some species of *Machaeroprosopus* also show elongated rostra [41].

Although the phylogenetic position of *Jupijkam paleofluvialis* differs based on the character, taxon set, and analysis type employed (Figs. 8, 9, 10 and 11), this species never groups together with other Norian-Rhaetian phytosaurs from North America. Thus, *Jupijkam paleofluvialis* demonstrates the persistence of several lineages of phytosaurs into the end-Triassic of northwestern Pangea. Although the holotype material of *Jupijkam paleofluvialis* was previously assigned to *Rutiodon* [72], *Jupijkam paleofluvialis* only groups with *Rutiodon carolinensis* in the Bayesian tip-dated phylogeny using the modified matrix of Datta and Ray [22] with very weak posterior branch support (< 0.5; Fig. 11a). These results support the naming of a new genus for the Nova Scotia phytosaur. The recognition of *Jupijkam paleofluvialis* as a distinct taxon highlights the problematic assignment of nearly all substantial phytosaur cranial material from eastern North America to *Rutiodon carolinensis* (e.g., [17, 18, 24, 72]) and suggests this region could harbor additional phytosaur diversity.

Both parsimony and Bayesian tip-dating analyses also support the placement of all Rhaetian western North American phytosaur species into a single genus, *Machaeroprosopus.* Phytosaur species placed in the genus *Redondasaurus* are invariably deeply nested within *Machaeroprosopus* in the phylogenetic trees generated in this study (Figs. 8 and 10a). Synonymy of *Redondasaurus* with *Machaeroprosopus* was most proposed by Hungerbühler et al. [41], who extensively reviewed the anatomy of all species of *Machaeroprosopus* and found *M. lottorum* bridged the morphological gap between species of *Machaeroprosopus* and *Redondasaurus*; this result is reflected especially clearly in the Bayesian tip-dated phylogeny of the Jones and Butler [45] matrix (Fig. 10a).

### Tempo of phytosaur diversification and extinction

Because phytosaur species show a well-established pattern of species-level turnover in regions where the fossil record of this clade is rich [58, 85], understanding the ages of phytosaur clades is essential for a better understanding of the timeline of faunal change throughout the Triassic. Although preliminary estimates of phytosaur divergence times have been made [22], this study is, to the author’s knowledge, the first to explicitly incorporate fossil age data to resolve the phylogeny of Phytosauria. Comparison of the Bayesian tip-dated trees generated using different character and taxon sets allows for robust inference of the tempo of phytosaur clade emergence (Figs. 10 and 11; Table 4). Both analyses support an Anisian-Ladinian age for the split at the base of Parasuchidae, followed by extensive diversification in throughout Carnian and regional diversifications in the northern hemisphere during the late Carnian and Norian, primarily within the clade containing *Parasuchus* spp. and relatives, the clade containing *Smilosuchus*, *Leptosuchus*, and *Nicrosaurus*, and within *Mystriosuchus* spp. Although the majority of species included in both analyses are from the northern hemisphere, biogeographic reconstructions suggest divergences of Carnian-Norian age occurred across Pangea (Figs. 10 and 11; Table 4).

**Table 4:** Number of dispersals between hemispheres compared for the two phylogenies under the DIVALIKE model

Time Period	Number of Dispersals, Matrix 1	Number of Dispersals, Matrix 2
Pre-Carnian	0	0 (4^b^)
Carnian	3	2
Norian	2^a^	1^a^
Rhaetian	0	0

^a^includes Carnian to Norian dispersals

^b^All of these related to nodes with unresolved hemispheric origin (e.g., India-Europe to India-Europe)

Recently, Datta and Ray [22] suggested that phytosaurs underwent an extinction event at the start of the Norian. This result is not supported for the major lineages of parasuchid phytosaurs by any of the trees generated in this study, which find that the major splits in the parasuchid tree span the Carnian-Norian. Although the record of *Parasuchus*-type phytosaurs appears to end in the early Norian, this is only a segment of the total diversity of the clade, and indeed further discoveries of one or two species resembling *Paleorhinus*, *Parasuchus*, or *Wannia* would be needed to break even this pattern (Figs. 10 and 11). Thus, the extinction of non-mystriosuchine phytosaurs proposed by Datta and Ray [22] is unsupported by explicit integration of morphological and temporal data to reconstruct the phylogenetic relationships of phytosaurs. Alternatively, the drop-off of early-diverging parasuchid fossils in the early Norian may be due to both the poorly- sampled nature of southern hemisphere phytosaur faunas where the diversity of this grade is concentrated [22, 45, 47] and the fluctuating taxonomy of species placed in the early-diverging phytosaur genera like *Paleorhinus* and *Parasuchus* (e.g., [13, 23, 47, 85, 87, 88]). However, detailed biostratigraphic analyses of phytosaurs and other reptiles from the American southwest do show that considerable turnover occurred among mystriosuchine -grade phytosaurs over the course of the Norian ([56, 58, 60, 75]; though see [77]). Indeed, whereas the evidence for early-diverging parasuchids suddenly going extinct during this period is ambiguous, mystriosuchine faunas in the northern hemisphere experienced considerable changes in their composition, a result reflected in the tip-dated Bayesian phylogenies generated in this paper (Figs. 10 and 11). These results highlight complex clade- and region-specific patterns of diversification that occurred among Norian phytosaurs.

By the end of the Norian, only a handful of western North American leptosuchomorph phytosaur clades continue to speciate (Figs. 10 and 11). Phytosaurs become exceptionally rare in the Late Triassic of southern Pangea (e.g., [2, 22, 85]) as only a handful of species persisted in the northern hemisphere (Figs. 10 and 11) following established turnover events that are important for using phytosaurs as markers of biochrons in western North America. The recognition of a new species of phytosaur, *Jupijkam paleofluvialis*, representing the northernmost extent of this clade (e.g., [22, 90]:Fig. 29, inset; [57]) highlights the northernmost extend of Pangea as an unsampled region of phytosaur diversity.

### Drivers of Pangean reptile biogeography

Throughout the Triassic, the floras and faunas of Pangaea experienced major climactic shifts that modified habitats across the continent. In particular, the periodic expansion of a desert belt across the middle of the supercontinent appears to have suppressed the dispersal of clades like dinosaurs [36, 48, 94]. In particular, the Carnian Pluvial Event appears to have been associated with the breakdown of this arid belt, facilitating the dispersal of dinosaurs and their closest relatives between hemispheres over a period of 5 to 10 million years [36]. However, whether similar patterns occur in other tetrapods has only been preliminarily investigated using phylogenies (e.g., [22, 27]). As such, stronger inference of barriers to tetrapod dispersal across Pangea requires that similar biogeographic signals are recorded in multiple distantly related clades.

Through conducting multiple phylogenetic analyses of different datasets under different optimality criteria and explicitly comparing different biogeographic models to reconstruct the historical biogeography of phytosaurs, I was able to robustly infer the presence of restrictions to trans-Pangean dispersal of this clade (Figs. 10 and 11; Table 4). Despite the topological differences of the strict consensus and maximum clade credibility trees generated using the two matrices employed in this study, all the phylogenetic analyses that I conducted strongly suggest an uptick in trans-hemispheric dispersal of phytosaur clades during the Carnian and early Norian (Table 4). This matches the biogeographic pattern inferred using phylogenies of early dinosaurs ([36]; also see reconstructions in [53, 59]).

By the middle of the Norian, trans-hemispheric dispersals of phytosaurs cease, and regionalized radiations occur in Europe and western North America (Figs. 10 and 11). Although additional fossils will be needed from the southern hemisphere to verify whether regional radiation was the primary driver of phytosaur diversity following the onset of the Norian, at least one phytosaur assemblage from the Carnian-Rhaetian of southern Pangea shows this pattern [22]. The identification of *Jupijkam paleofluvialis* as a distinct taxon that may be only distantly related to other eastern North American phytosaurs adds to the evidence for regional endemism as a driver of phytosaur diversity in the Norian-Rhaetian following the reestablishment of the arid central Pangean belt. Regional endemicity is well-established in other vertebrate clades known from the Central Atlantic rift valleys, especially semionotid fishes [63–66, 71]. Together with previous studies of other trans-Pangean non-dinosaur faunas [93], the results presented in this study add support for the hypothesis that periodic arid belts were an important mechanism structuring Pangean tetrapod biogeography.

## Conclusions

- 1. Here, I have described a new genus and species of phytosaur, *Jupijkam paleofluvialis* gen. et sp. nov., from the uppermost Norian White Water Member of the Blomidon Formation in Nova Scotia, Canada. The holotype specimen, collected in 1974, includes a partial skull and fragmentary postcrania, including a single complete osteoderm.
- 2. Multiple autapomorphies, including a deeply invaginated, anteroposteriorly-running groove along the lateral surface of the rostrum that terminates posterior of the anterior corner of the antorbital fenestra, a diastema between the four anteriormost premaxillary teeth and the rest of the tooth row, the absence of ornamentation on rostrum besides occasional foramina, and a primarily laterally-facing paranasal, clearly distinguish *Jupijkam paleofluvialis* from other phytosaurs, including the far older *Rutiodon carolinensis*.
- 3. *Jupijkam paleofluvialis* is resolved as a mystriosuchine phytosaur outside Leptosuchomorpha, although its precise affinities differ based on analysis type and matrix used. There is no strong evidence that eastern North American phytosaurs (*Jupijkam paleofluvialis*, *Rutiodon carolinensis*) form a clade, indicating regional provincialism along the early Atlantic rift.
- 4. Biogeographic analyses of the tip-dated Bayesian phylogenies of phytosaurs generated using two different datasets strongly suggest an uptick in trans-hemispheric migration or dispersal during the Carnian and early Norian, with the origin of phytosaurs placed in the northern hemisphere.
- 5. An increase in trans-hemispheric dispersal of phytosaurs during the Carnian, and the absence of such dispersals immediately preceding or following this period, matches biogeographic patterns seen in other Triassic tetrapods, especially dinosaurs. Thus, phytosaur phylogeny and historical biogeography support a model of Pangean biogeography whereby the distributions and dispersal capabilities of reptiles between the northern and southern hemisphere were restricted by the formation and disappearance of an arid belt across central Pangea.

## Supplementary Information

**Additional file 1. **

## Data Availability

All data generated for this study is provided in the manuscript and supplementary information to this article. The fossil specimens examined are catalogued in the vertebrate paleontology collection of the Yale Peabody Museum of Natural History, a licensed public repository located in New Haven, Connecticut, USA. The associated LSID for this manuscript is urn:lsid:zoobank.org:pub:59607221-CAA1-4770-9305-7C7450F8529F.

## Abbreviations

TTU-P: Texas Tech University, Lubbock, Texas, USA
USNM VP: United States National Museum of Natural History Vertebrate Paleontology collections, Washington DC, USA
YPM VPPU: Princeton University Vertebrate Paleontology Collections in the Yale Peabody Museum of Natural History, New Haven, CT, USA
