# Supplementary material for: A late-surviving phytosaur from the northern Atlantic rift reveals climate constraints on Triassic reptile biogeography
Source: BMC Ecol Evol. 2023 Jul 17;23:33. doi: 10.1186/s12862-023-02136-8 (PMC10351158; doi:10.1186/s12862-023-02136-8)
Supplement: Supplementary file 1 — Additional file 1. [file 12862_2023_2136_MOESM1_ESM.zip › Parsimony_matrices_and_results.zip!/Parsimony/Phytosaur_apomorphies.docx]

MATRIX 1:

2 trees found of length 315, CI 0.403, RI 0.654. The following character states are in TNT format, such that character 0 below=character 1 in the matrix, etc.

Diandongosuchus_fuyuanensis :

No autapomorphies

Angistorhinus_grandis :

Char. 11: 0 → 1

Char. 16: 0 → 1

Char. 62: 1 → 0

Char. 65: 0 → 1

Angistorhinus_talainti :

Char. 75: 0 → 1

Coburgosuchus_goeckeli :

Char. 49: 0 → 1

Ebrachosuchus_neukami :

Char. 0: 0 → 1

Char. 7: 0 → 2

Char. 10: 0 → 2

Char. 21: 1 → 2

Char. 22: 1 → 0

Char. 33: 0 → 1

Char. 41: 1 → 0

Char. 53: 1 → 0

Char. 67: 1 → 2

Char. 72: 0 → 1

Char. 82: 1 → 3

Char. 86: 0 → 2

Char. 88: 0 → 1

Leptosuchus_crosbiensis :

Char. 47: 0 → 1

Leptosuchus_studeri :

Char. 21: 3 → 2

Char. 49: 0 → 1

Machaeroprosopus_andersoni :

Char. 6: 2 → 4

Char. 59: 2 → 1

Machaeroprosopus_buceros :

Char. 6: 1 → 4

Char. 78: 0 → 1

Machaeroprosopus_jablonskiae :

No autapomorphies

Machaeroprosopus_lottorum :

Char. 58: 2 → 3

Char. 69: 1 → 0

Machaeroprosopus_mccauleyi :

Char. 4: 0 → 1

Char. 24: 0 → 1

Char. 69: 1 → 0

Machaeroprosopus_pristinus :

Char. 10: 1 → 2

Machaeroprosopus_zunii :

Char. 37: 3 → 1

Char. 47: 0 → 1

Mystriosuchus_planirostris :

Char. 32: 2 → 0

Mystriosuchus_westphali :

Char. 5: 0 → 2

Char. 6: 1 → 0

Char. 29: 1 → 0

Char. 61: 0 → 1

Mystriosuchus_steinbergeri :

Char. 18: 1 → 0

Char. 72: 2 → 1

Nicrosaurus_kapffi :

Char. 37: 3 → 2

Char. 65: 0 → 1

Char. 68: 2 → 1

Char. 70: 1 → 0

Char. 78: 0 → 1

Nicrosaurus_meyeri :

Char. 4: 1 → 0

Char. 42: 2 → 0

Paleorhinus_sawini :

Char. 4: 0 → 1

Char. 17: 0 → 1

Char. 21: 1 → 3

Char. 24: 0 → 1

Char. 27: 1 → 0

Char. 35: 0 → 1

Char. 59: 0 → 1

Char. 65: 0 → 2

Parasuchus_angustifrons :

Char. 9: 0 → 1

Char. 68: 1 → 0

Parasuchus_bransoni :

Char. 18: 0 → 1

Char. 70: 1 → 0

Parasuchus_hislopi :

Char. 26: 0 → 1

Char. 35: 1 → 2

Pravusuchus_hortus :

Char. 4: 1 → 0

Char. 20: 0 → 1

Char. 29: 1 → 0

Char. 32: 2 → 1

Char. 37: 3 → 1

Char. 86: 0 → 1

Protome_batalaria :

Char. 32: 2 → 1

Char. 93: 0 → 1

Redondasaurus_bermani :

Char. 53: 0 → 1

Redondasaurus_gregorii :

Char. 88: 0 → 1

Rutiodon_carolinensis :

No autapomorphies

Smilosuchus_adamanensis :

No autapomorphies

Smilosuchus_gregorii :

Char. 2: 0 → 1

Char. 3: 0 → 1

Smilosuchus_lithodendrorum :

Char. 2: 0 → 1

Char. 42: 2 → 01

Char. 56: 1 → 0

Wannia_scurriensis :

Char. 82: 1 → 2

Volcanosuchus_statisticae :

Char. 2: 0 → 1

Char. 6: 1 → 2

Colossosuchus_techniensis :

Char. 2: 0 → 1

Char. 14: 1 → 0

Char. 15: 1 → 0

Char. 21: 1 → 0

Char. 24: 0 → 1

Char. 31: 0 → 1

Char. 32: 0 → 2

Char. 35: 0 → 1

Char. 36: 0 → 1

Char. 51: 1 → 0

Char. 56: 0 → 1

Char. 59: 0 → 1

Char. 66: 0 → 1

Char. 70: 1 → 0

Char. 78: 0 → 1

Char. 89: 0 → 1

Char. 93: 0 → 1

Arganarhinus_magnoculus :

Char. 4: 0 → 1

Char. 6: 0 → 1

Char. 10: 0 → 1

Char. 22: 0 → 1

Char. 80: 0 → 2

Char. 86: 0 → 1

Char. 87: 1 → 0

Jupijkam_paleofluvialis :

Char. 2: 0 → 1

Char. 3: 0 → 1

Node 37 :

Char. 4: 0 → 1

Char. 57: 0 → 1

Char. 91: 0 → 1

Node 38 :

Char. 6: 0 → 1

Char. 13: 1 → 2

Char. 18: 0 → 1

Char. 33: 0 → 1

Char. 37: 0 → 2

Char. 80: 0 → 2

Node 39 :

Char. 0: 0 → 1

Char. 82: 1 → 0

Node 40 :

Char. 7: 1 → 0

Char. 15: 0 → 1

Char. 49: 0 → 1

Char. 54: 0 → 1

Node 41 :

No synapomorphies

Node 42 :

Char. 56: 1 → 2

Node 43 :

Char. 4: 0 → 1

Char. 9: 0 → 1

Char. 18: 1 → 0

Char. 24: 0 → 1

Char. 42: 0 → 2

Char. 63: 1 → 0

Char. 72: 2 → 1

Char. 80: 2 → 1

Node 44 :

Char. 10: 2 → 1

Char. 15: 1 → 0

Char. 17: 0 → 1

Char. 21: 1 → 3

Char. 89: 0 → 1

Node 45 :

Char. 40: 0 → 1

Char. 48: 0 → 2

Char. 52: 2 → 1

Char. 53: 1 → 0

Char. 56: 0 → 1

Char. 77: 1 → 0

Node 46 :

Char. 10: 0 → 2

Char. 41: 1 → 0

Char. 49: 1 → 0

Char. 55: 1 → 0

Char. 74: 0 → 1

Node 47 :

Char. 59: 0 → 1

Node 48 :

Char. 81: 0 → 1

Node 49 :

Char. 22: 0 → 1

Char. 35: 0 → 1

Node 50 :

Char. 55: 1 → 0

Node 51 :

Char. 6: 2 → 4

Char. 28: 0 → 1

Char. 32: 2 → 0

Node 52 :

Char. 66: 2 → 0

Node 53 :

Char. 8: 0 → 1

Char. 52: 1 → 2

Char. 53: 0 → 1

Char. 74: 1 → 0

Node 54 :

Char. 2: 0 → 1

Char. 74: 1 → 0

Node 55 :

Char. 3: 0 → 1

Char. 6: 1 → 2

Char. 86: 0 → 2

Node 56 :

Char. 8: 0 → 1

Char. 46: 0 → 1

Char. 56: 1 → 2

Char. 58: 1 → 2

Node 57 :

Char. 28: 0 → 1

Char. 60: 0 → 2

Char. 64: 0 → 1

Char. 69: 0 → 1

Node 58 :

Char. 37: 3 → 1

Char. 47: 0 → 1

Char. 86: 0 → 1

Node 59 :

Char. 21: 3 → 2

Char. 39: 1 → 0

Node 60 :

Char. 52: 1 → 0

Node 61 :

Char. 19: 1 → 0

Char. 45: 1 → 0

Char. 89: 1 → 0

Node 62 :

Char. 21: 1 → 2

Node 63 :

Char. 1: 1 → 2

Char. 11: 0 → 1

Char. 86: 0 → 2

Node 64 :

Char. 32: 2 → 0

Char. 52: 1 → 0

Node 65 :

Char. 52: 1 → 2

Char. 56: 2 → 3

Char. 58: 2 → 3

Char. 66: 2 → 0

Node 66 :

Char. 8: 0 → 1

Char. 86: 0 → 1

Char. 91: 0 → 1

Node 67 :

Char. 18: 0 → 1

Char. 32: 2 → 1

MATRIX 2:

27 trees found of length 349, CI = 0.444, RI = 0.576

Tree 27 :

Diandongosuchus_fuyuanensis_237 :

No autapomorphies

Wannia_scurriensis_225.0 :

Char. 4: 0 → 1

Char. 6: 0 → 1

Char. 7: 0 → 1

Char. 8: 2 → 1

Char. 14: 0 → 1

Char. 27: 1 → 0

Char. 38: 2 → 1

Char. 44: 2 → 1

Char. 49: 2 → 1

Char. 50: 0 → 1

Char. 70: 0 → 1

Char. 73: 0 → 2

Char. 80: 1 → 0

Char. 82: 1 → 0

Parasuchus_hislopi_225.0 :

Char. 16: 0 → 1

Char. 23: 0 → 1

Char. 42: 1 → 0

Char. 71: 1 → 0

Char. 77: 0 → 2

Char. 79: 0 → 1

Parasuchus_bransoni_225.0 :

Char. 2: 1 → 0

Char. 8: 2 → 0

Char. 24: 0 → 1

Char. 26: 0 → 1

Char. 28: 1 → 2

Char. 30: 0 → 1

Char. 44: 2 → 1

Char. 49: 2 → 0

Char. 71: 1 → 0

Char. 72: 0 → 1

Char. 82: 1 → 0

Parasuchus_angustifrons_228.0 :

Char. 9: 0 → 1

Char. 18: 0 → 1

Char. 30: 0 → 1

Char. 41: 0 → 1

Char. 43: 1 → 2

Char. 70: 0 → 1

Char. 73: 0 → 1

Ebrachosuchus_neukami_228.0 :

Char. 0: 12 → 3

Char. 22: 0 → 1

Char. 44: 2 → 3

Char. 54: 1 → 0

Char. 70: 0 → 2

Char. 72: 0 → 2

Char. 73: 0 → 3

Char. 75: 1 → 0

Char. 86: 1 → 0

Char. 89: 0 → 1

Char. 105: 0 → 1

Brachysuchus_megalodon_228.5 :

Char. 2: 1 → 0

Char. 41: 0 → 1

Char. 59: 0 → 1

Char. 92: 1 → 2

Angistorhinus_grandis_225.0 :

Char. 30: 0 → 1

Char. 40: 1 → 0

Rutiodon_carolinensis_218.0 :

Char. 1: 1 → 0

Char. 40: 1 → 0

Char. 43: 02 → 1

Protome_batalaria_218.0 :

Char. 31: 2 → 1

Char. 40: 1 → 0

Char. 49: 3 → 0

Char. 50: 1 → 0

Char. 51: 1 → 0

Char. 91: 0 → 1

Pravusuchus_hortus_217.0 :

Char. 13: 1 → 0

Char. 30: 0 → 1

Char. 40: 1 → 0

Char. 43: 2 → 1

Char. 48: 0 → 1

Char. 63: 0 → 1

Char. 70: 2 → 0

Char. 74: 0 → 1

Char. 77: 2 → 1

Leptosuchus_crosbiensis_220.0 :

Char. 22: 1 → 0

Char. 61: 1 → 0

Smilosuchus_adamenensis_220.0 :

Char. 46: 0 → 1

Char. 77: 2 → 0

Char. 84: 1 → 0

Char. 99: 0 → 1

Smilosuchus_gregorii_220.0 :

Char. 4: 2 → 0

Char. 5: 0 → 1

Char. 37: 0 → 1

Char. 44: 1 → 3

Char. 72: 0 → 2

Char. 94: 1 → 2

Machaeroprosopus_jabolonskiae_2 :

Char. 13: 0 → 1

Char. 40: 1 → 0

Char. 59: 2 → 1

Char. 105: 2 → 0

Machaeroprosopus_lottorum_208.0 :

Char. 5: 0 → 1

Char. 8: 2 → 3

Char. 14: 1 → 0

Char. 16: 0 → 1

Char. 31: 2 → 1

Char. 41: 1 → 0

Char. 44: 1 → 3

Char. 52: 1 → 0

Char. 64: 0 → 1

Char. 65: 1 → 0

Char. 72: 0 → 2

Char. 79: 0 → 1

Char. 81: 1 → 0

Char. 103: 0 → 1

Machaeroprosopus_mccauleyi_207. :

Char. 5: 0 → 1

Char. 19: 1 → 0

Char. 49: 3 → 2

Char. 56: 0 → 2

Char. 60: 02 → 1

Char. 73: 0 → 3

Mystriosuchus_westphali_209.0 :

Char. 33: 0 → 1

Char. 44: 1 → 2

Char. 52: 1 → 0

Mystriosuchus_planirostris_209. :

Char. 8: 2 → 3

Char. 16: 0 → 1

Char. 31: 2 → 1

Char. 41: 1 → 0

Char. 42: 0 → 1

Char. 43: 2 → 3

Char. 103: 0 → 1

Volcanosuchus_statisticae_227.0 :

Char. 2: 1 → 0

Char. 12: 2 → 1

Char. 35: 0 → 1

Char. 38: 2 → 1

Char. 40: 1 → 2

Char. 47: 1 → 0

Char. 50: 1 → 2

Char. 51: 1 → 2

Char. 53: 1 → 2

Char. 71: 0 → 2

Char. 72: 0 → 3

Char. 73: 0 → 2

Colossosuchus_techniensis_227.0 :

Char. 3: 1 → 2

Char. 7: 0 → 1

Char. 25: 1 → 0

Char. 31: 0 → 2

Char. 33: 0 → 1

Char. 37: 0 → 1

Char. 40: 1 → 2

Char. 43: 0 → 1

Char. 80: 2 → 1

Char. 93: 1 → 2

ISIR276_Indian_phytosaur_sp1_22 :

Char. 40: 1 → 0

Char. 56: 0 → 3

ISIR271_Indian_phytosaur_sp2_22 :

Char. 15: 1 → 2

Char. 26: 0 → 1

Char. 44: 2 → 3

Char. 61: 0 → 1

Char. 77: 2 → 0

Char. 80: 2 → 0

Jupijkam_paleofluvialis_208.5 :

Char. 5: 0 → 1

Char. 42: 1 → 0

Char. 48: 0 → 1

Char. 49: 3 → 1

Node 25 :

No synapomorphies

Node 26 :

Char. 2: 1 → 0

Char. 7: 0 → 1

Char. 25: 1 → 0

Char. 40: 1 → 0

Char. 72: 0 → 1

Node 27 :

Char. 16: 0 → 1

Char. 17: 1 → 0

Char. 39: 0 → 2

Char. 69: 0 → 1

Char. 79: 0 → 1

Char. 89: 0 → 1

Node 28 :

Char. 10: 1 → 2

Char. 12: 0 → 2

Char. 18: 0 → 1

Char. 22: 0 → 1

Char. 43: 1 → 02

Char. 60: 0 → 1

Char. 65: 0 → 1

Char. 71: 1 → 0

Char. 76: 0 → 2

Char. 77: 0 → 2

Char. 80: 1 → 2

Char. 84: 0 → 1

Char. 98: 0 → 1

Char. 103: 0 → 1

Node 29 :

Char. 8: 2 → 3

Char. 16: 0 → 1

Node 30 :

Char. 26: 0 → 1

Char. 27: 1 → 2

Char. 60: 1 → 2

Char. 62: 1 → 0

Char. 86: 1 → 0

Char. 102: 1 → 2

Node 31 :

Char. 20: 0 → 1

Char. 46: 1 → 0

Char. 49: 2 → 3

Char. 55: 0 → 1

Char. 66: 0 → 1

Char. 67: 0 → 1

Char. 78: 0 → 1

Char. 100: 0 → 1

Char. 105: 0 → 1

Node 32 :

Char. 14: 0 → 1

Char. 15: 1 → 2

Char. 21: 0 → 1

Char. 25: 1 → 0

Char. 28: 1 → 2

Char. 31: 1 → 2

Char. 34: 2 → 0

Char. 41: 0 → 1

Char. 44: 2 → 1

Char. 61: 0 → 1

Char. 93: 1 → 2

Node 33 :

Char. 13: 1 → 0

Char. 56: 1 → 0

Char. 100: 1 → 0

Char. 103: 1 → 0

Node 34 :

Char. 20: 1 → 2

Char. 36: 1 → 2

Char. 37: 0 → 1

Char. 57: 1 → 2

Char. 74: 0 → 1

Node 35 :

Char. 21: 2 → 1

Char. 25: 0 → 1

Char. 49: 3 → 0

Char. 50: 1 → 2

Char. 57: 2 → 1

Char. 64: 0 → 1

Char. 77: 2 → 0

Char. 88: 1 → 2

Char. 89: 0 → 2

Char. 93: 2 → 1

Node 36 :

Char. 8: 2 → 0

Char. 31: 1 → 0

Char. 34: 2 → 1

Char. 51: 1 → 3

Char. 63: 0 → 2

Char. 85: 0 → 1

Char. 88: 01 → 2

Char. 89: 0 → 2

Char. 103: 1 → 2

Char. 104: 0 → 1
