# Supplementary material for: A late-surviving phytosaur from the northern Atlantic rift reveals climate constraints on Triassic reptile biogeography
Source: BMC Ecol Evol. 2023 Jul 17;23:33. doi: 10.1186/s12862-023-02136-8 (PMC10351158; doi:10.1186/s12862-023-02136-8)
Supplement: Supplementary file 1 — Additional file 1. [file 12862_2023_2136_MOESM1_ESM.zip › Biogeobears_matrix_1.zip!/Biogeobears_matrix_1/PDF/Rplot10.pdf]

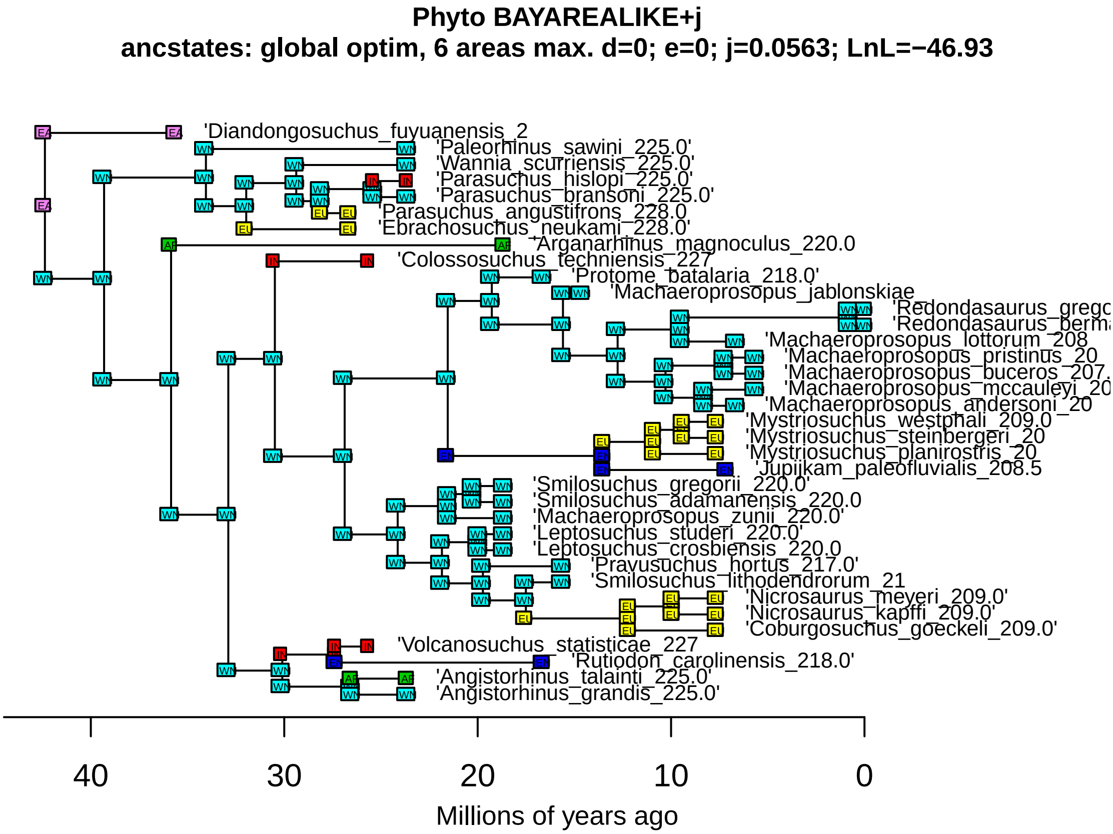
Phyto BAYAREALIKE+j
ancstates: global optim, 6 areas max. d=0; e=0; j=0.0563; LnL=−46.93
'Diandongosuchus_fuyuanensis_2
'Paleorhinus_sawini_225.0'
'Wannia_scurriensis_225.0'
'Parasuchus_hislopi_225.0'
'Parasuchus_bransoni_225.0'
'Parasuchus_angustifrons_228.0
'Ebrachosuchus_neukami_228.0'
'Arganarhinus_magnoculus_220.0
'Colossosuchus_techniensis_227
'Protome_batalaria_218.0'
'Machaeroprosopus_jablonskiae
'Machaeroprosopus_lottorum_208
'Machaeroprosopus_pristinus_20
'Machaeroprosopus_buceros_207.
'Machaeroprosopus_mccauleyi_20
'Machaeroprosopus_andersoni_20
'Mystriosuchus_westphali_209.0
'Mystriosuchus_steinbergeri_20
'Mystriosuchus_planirostris_20
'Jupijkam_paleofluvialis_208.5
'Smilosuchus_gregorii_220.0'
'Smilosuchus_adamanensis_220.0
'Machaeroprosopus_zunii_220.0'
'Leptosuchus_studeri_220.0'
'Leptosuchus_crosbiensis_220.0
'Pravusuchus_hortus_217.0'
'Smilosuchus_lithodendrorum_21
'Nicrosaurus_meyeri_209.0'
'Nicrosaurus_kapffi_209.0'
'Coburgosuchus_goeckeli_209.0'
'Volcanosuchus_statisticae_227
'Rutiodon_carolinensis_218.0'
'Angistorhinus_talainti_225.0'
'Angistorhinus_grandis_225.0'
40
30
20
10
0
Millions of years ago
