# Supplementary material for: A late-surviving phytosaur from the northern Atlantic rift reveals climate constraints on Triassic reptile biogeography
Source: BMC Ecol Evol. 2023 Jul 17;23:33. doi: 10.1186/s12862-023-02136-8 (PMC10351158; doi:10.1186/s12862-023-02136-8)

Phyto BAYAREALIKE+j

ancstates: global optim, 6 areas max. d=0; e=0; j=0.0563; LnL=−46.93

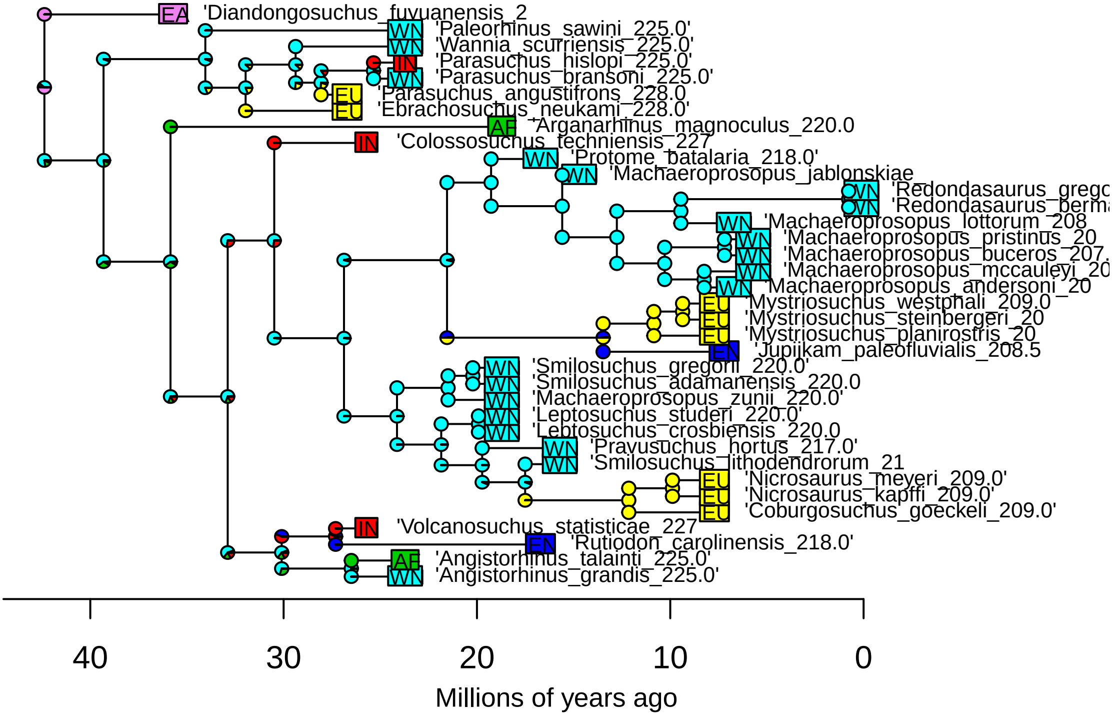

Supplement: Supplementary file 1 — Additional file 1. [file 12862_2023_2136_MOESM1_ESM.zip › Biogeobears_matrix_1.zip!/Biogeobears_matrix_1/PDF/Rplot11.pdf]
