# Supplementary material for: A late-surviving phytosaur from the northern Atlantic rift reveals climate constraints on Triassic reptile biogeography
Source: BMC Ecol Evol. 2023 Jul 17;23:33. doi: 10.1186/s12862-023-02136-8 (PMC10351158; doi:10.1186/s12862-023-02136-8)
Supplement: Supplementary file 1 — Additional file 1. [file 12862_2023_2136_MOESM1_ESM.zip › Biogeobears_matrix_1.zip!/Biogeobears_matrix_1/PDF/Rplot01.pdf]

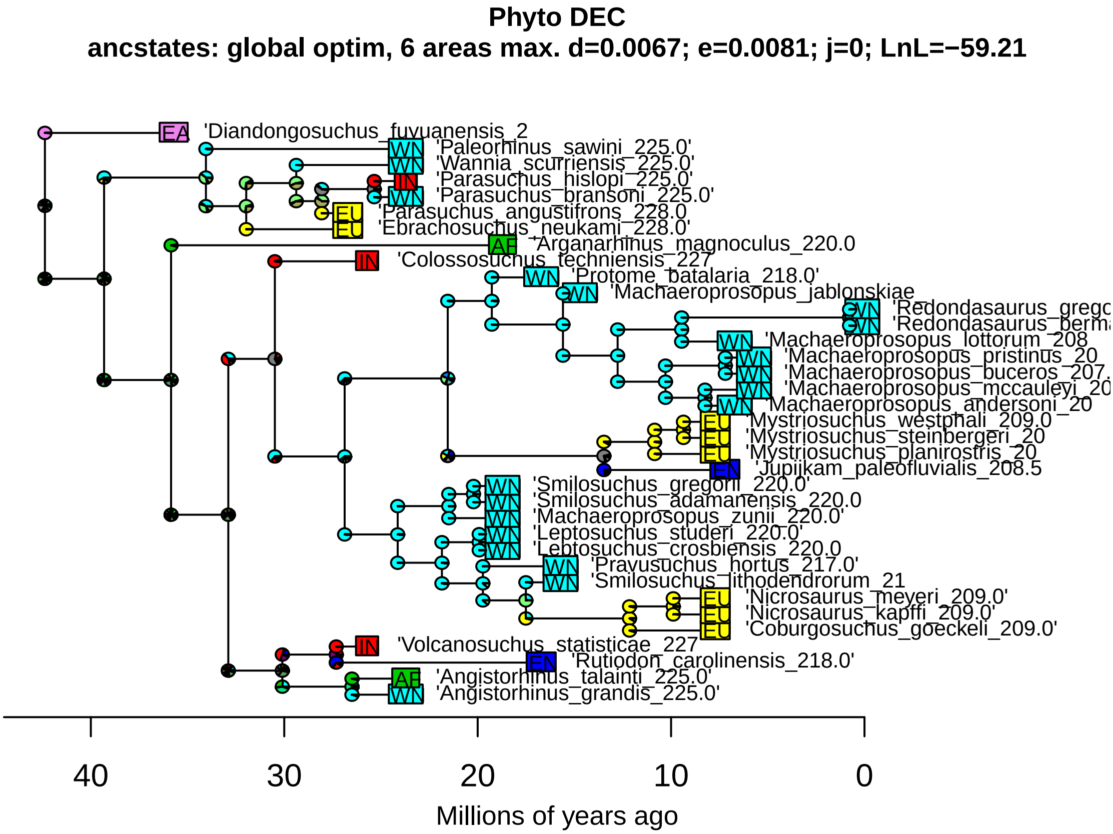
Phyto DEC
ancstates: global optim, 6 areas max. d=0.0067; e=0.0081; j=0; LnL=−59.21
EA 'Diandongosuchus_fuyuanensis_2
WN 'Paleorhinus_sawini_225.0'
WN 'Wannia_scurriensis_225.0'
IN 'Parasuchus_hislopi_225.0'
WN 'Parasuchus_bransoni_225.0'
EU 'Parasuchus_angustifrons_228.0
EU 'Ebrachosuchus_neukami_228.0'
AF 'Arganarhinus_magnoculus_220.0
IN 'Colossosuchus_techniensis_227
WN 'Protome_batalaria_218.0'
WN 'Machaeroprosopus_jablonskiae
WN 'Redondasaurus_grego
WN 'Redondasaurus_berma
WN 'Machaeroprosopus_lottorum_208
WN 'Machaeroprosopus_pristinus_20
WN 'Machaeroprosopus_buceros_207.
WN 'Machaeroprosopus_mccauleyi_20
WN 'Machaeroprosopus_andersoni_20
EU 'Mystriosuchus_westphali_209.0
EU 'Mystriosuchus_steinbergeri_20
EU 'Mystriosuchus_planirostris_20
EN 'Jupijkam_paleofluvialis_208.5
WN 'Smilosuchus_gregorii_220.0'
WN 'Smilosuchus_adamanensis_220.0
WN 'Machaeroprosopus_zunii_220.0'
WN 'Leptosuchus_studeri_220.0'
WN 'Leptosuchus_crosbiensis_220.0
WN 'Pravusuchus_hortus_217.0'
WN 'Smilosuchus_lithodendrorum_21
EU 'Nicrosaurus_meyeri_209.0'
EU 'Nicrosaurus_kapffi_209.0'
EU 'Coburgosuchus_goeckeli_209.0'
IN 'Volcanosuchus_statisticae_227
EN 'Rutiodon_carolinensis_218.0'
AF 'Angistorhinus_talainti_225.0'
WN 'Angistorhinus_grandis_225.0'
40
30
20
10
0
Millions of years ago
