# Supplementary material for: A late-surviving phytosaur from the northern Atlantic rift reveals climate constraints on Triassic reptile biogeography
Source: BMC Ecol Evol. 2023 Jul 17;23:33. doi: 10.1186/s12862-023-02136-8 (PMC10351158; doi:10.1186/s12862-023-02136-8)
Supplement: Supplementary file 1 — Additional file 1. [file 12862_2023_2136_MOESM1_ESM.zip › Biogeobears_matrix_2.zip!/Biogeobears_matrix_2/PDF/Rplot03.pdf]

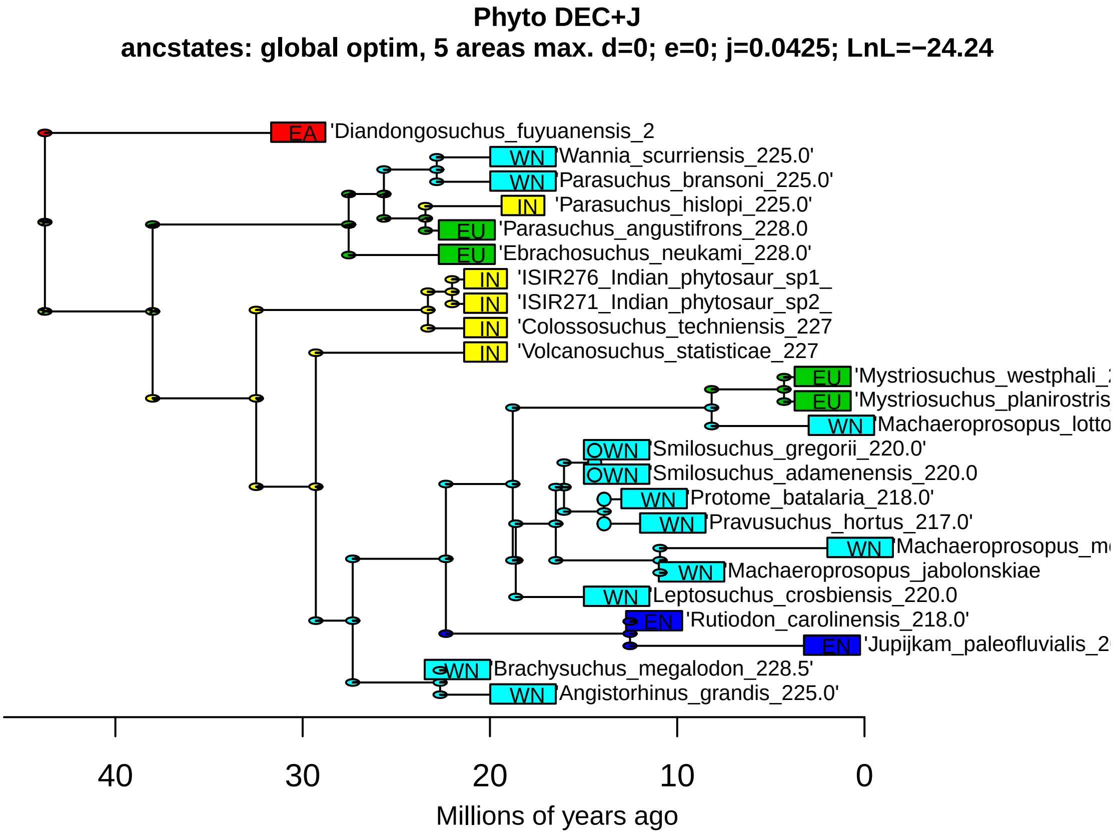
Phyto DEC+J
ancstates: global optim, 5 areas max. d=0; e=0; j=0.0425; LnL=−24.24
EA 'Diandongosuchus_fuyuanensis_2
WN 'Wannia_scurriensis_225.0'
WN 'Parasuchus_bransoni_225.0'
IN 'Parasuchus_hislopi_225.0'
EU 'Parasuchus_angustifrons_228.0
EU 'Ebrachosuchus_neukami_228.0'
IN 'ISIR276_Indian_phytosaur_sp1_
IN 'ISIR271_Indian_phytosaur_sp2_
IN 'Colossosuchus_techniensis_227
IN 'Volcanosuchus_statisticae_227
EU 'Mystriosuchus_westphali_
EU 'Mystriosuchus_planirostris
WN 'Machaeroprosopus_lotto
OWN 'Smilosuchus_gregorii_220.0'
OWN 'Smilosuchus_adamenensis_220.0
WN 'Protome_batalaria_218.0'
WN 'Pravusuchus_hortus_217.0'
WN 'Machaeroprosopus_m
WN 'Machaeroprosopus_jabolonskiae
WN 'Leptosuchus_crosbiensis_220.0
EN 'Rutiodon_carolinensis_218.0'
EN 'Jupijkam_paleofluvialis_2
WN 'Brachysuchus_megalodon_228.5'
WN 'Angistorhinus_grandis_225.0'
40
30
20
10
0
Millions of years ago
