# Supplementary material for: A late-surviving phytosaur from the northern Atlantic rift reveals climate constraints on Triassic reptile biogeography
Source: BMC Ecol Evol. 2023 Jul 17;23:33. doi: 10.1186/s12862-023-02136-8 (PMC10351158; doi:10.1186/s12862-023-02136-8)
Supplement: Supplementary file 1 — Additional file 1. [file 12862_2023_2136_MOESM1_ESM.zip › Biogeobears_matrix_2.zip!/Biogeobears_matrix_2/PDF/Rplot08.pdf]

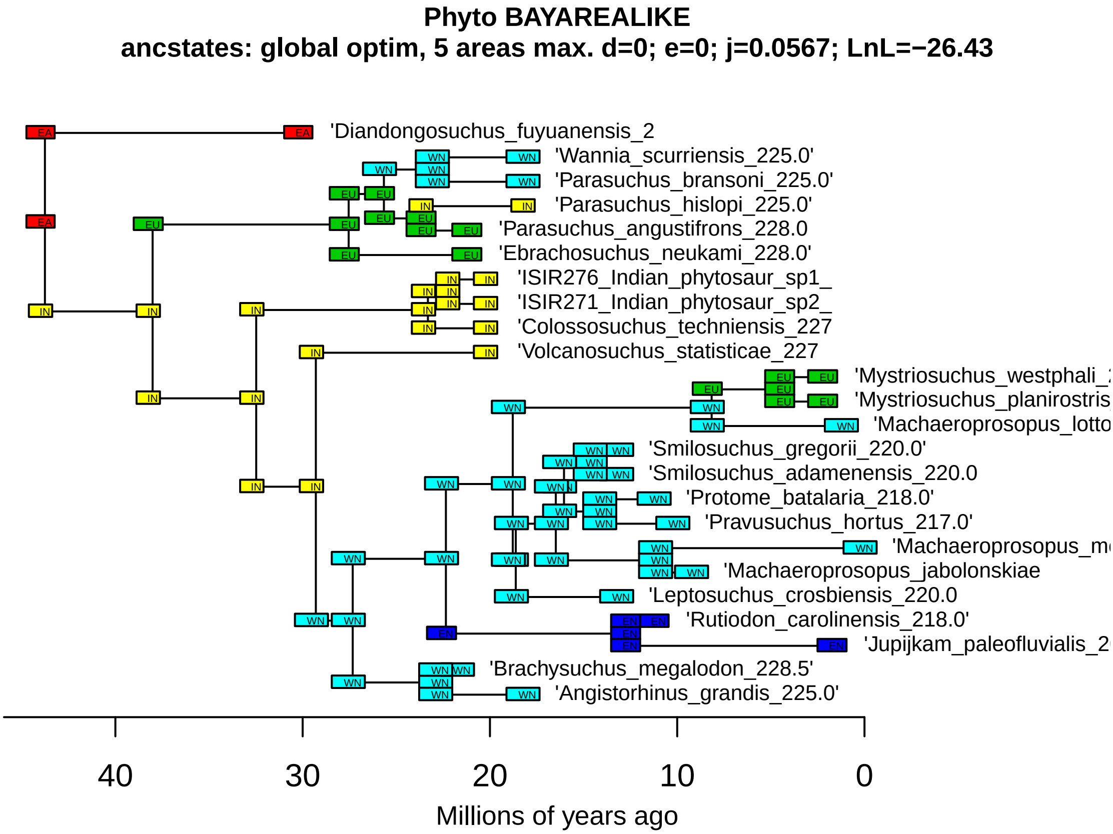
Phyto BAYAREALIKE
ancstates: global optim, 5 areas max. d=0; e=0; j=0.0567; LnL=−26.43
'Diandongosuchus_fuyuanensis_2
'Wannia_scurriensis_225.0'
'Parasuchus_bransoni_225.0'
'Parasuchus_hislopi_225.0'
'Parasuchus_angustifrons_228.0
'Ebrachosuchus_neukami_228.0'
'ISIR276_Indian_phytosaur_sp1_
'ISIR271_Indian_phytosaur_sp2_
'Colossosuchus_techniensis_227
'Volcanosuchus_statisticae_227
'Mystriosuchus_westphali_
'Mystriosuchus_planirostris
'Machaeroprosopus_lotto
'Smilosuchus_gregorii_220.0'
'Smilosuchus_adamenensis_220.0
'Protome_batalaria_218.0'
'Pravusuchus_hortus_217.0'
'Machaeroprosopus_m
'Machaeroprosopus_jabolonskiae
'Leptosuchus_crosbiensis_220.0
'Rutiodon_carolinensis_218.0'
'Jupijkam_paleofluvialis_2
'Brachysuchus_megalodon_228.5'
'Angistorhinus_grandis_225.0'
EA
EU
IN
WN
EN
40
30
20
10
0
Millions of years ago
